# New diphenylphosphane derivatives of ketoconazole are promising antifungal agents

**DOI:** 10.1038/s41598-019-52525-7

**Published:** 2019-11-07

**Authors:** Rodrigo F. M. de Almeida, Filipa C. Santos, Krzysztof Marycz, Michalina Alicka, Anna Krasowska, Jakub Suchodolski, Jarosław J. Panek, Aneta Jezierska, Radosław Starosta

**Affiliations:** 1 Centro de Química e Bioquímica, Departamento de Química e Bioquímica, Faculdade de Ciências da Universidade de Lisboa, Campo Grande, 1749-016 Lisboa Portugal; 2 Department of Experimental Biology, Faculty of Biology and Animal Science, Wroclaw University of Environmental and Life Sciences, Norwida 27B, 50-375 Wroclaw, Poland; 3 Faculty of Biotechnology, University of Wroclaw, F. Joliot-Curie 14a, 50-383 Wroclaw, Poland; 4 Faculty of Chemistry, University of Wroclaw, F. Joliot-Curie 14, 50-383 Wroclaw, Poland

**Keywords:** Drug discovery and development, Antifungal agents

## Abstract

Four new derivatives of ketoconazole (**Ke**) were synthesized: diphenylphosphane (**KeP**), and phosphane chalcogenides: oxide (**KeOP**), sulphide (**KeSP**) and selenide (**KeSeP**). These compounds proved to be promising antifungal compounds towards *Saccharomyces cerevisiae* and *Candida albicans*, especially in synergy with fluconazole. Simulations of docking to the cytochrome P450 14α-demethylase (azoles’ primary molecular target) proved that the new **Ke** derivatives are capable of inhibiting this enzyme by binding to the active site. Cytotoxicity towards hACSs (human adipose-derived stromal cells) of the individual compounds was studied and the IC_50_ values were higher than the MIC_50_ for *C*. *albicans* and *S*. *cerevisiae*. **KeP** and **KeOP** increased the level of the p21 gene transcript but did not change the level of p53 gene transcript, a major regulator of apoptosis, and decreased the mitochondrial membrane potential. Taken together, the results advocate that the new ketoconazole derivatives have a similar mechanism of action and block the lanosterol 14α-demethylase and thus inhibit the production of ergosterol in *C*. *albicans* membranes.

## Introduction

In recent times, the emergence of pathogenic fungi resistant to commonly used antifungal drugs has been occurring at unprecedented rates^1^. Antifungal resistance is especially a problem with *Candida* infections, and *Candida albicans* is the predominant cause of invasive fungal infections in humans^2^, responsible for ~15% of nosocomial sepsis^3^. Invasive candidiases are mortal in 50–80% rates^4,5^, reaching ~70% even among patients undergoing antifungal therapy^6,7^. Thus, the search for new antifungals and alternative therapies has become increasingly important. To tackle this problem from a medicinal chemistry perspective, and considering that a modification of existing drugs is more straightforward than developing a new class of therapeutic agents, we chose ketoconazole (**Ke**), a synthetic imidazole antifungal drug approved by FDA in 1981^8^, as a starting molecule for our present study. **Ke** was, for the following decade, the only antifungal available for oral treatment of systemic fungal infections caused by pathogenic yeasts. Its mechanism of action is well established. Similarly to other azole based drugs, its primary molecular target is cytochrome P450 14-alpha-demethylase (P45014DM)^9–15^, acting as a competitive inhibitor. **Ke** binds the Fe atom of cytochrome P450 through the N atom of its imidazole ring. Still in the 1980’s the interest on **Ke** expanded to other areas, namely as a potential anticancer agent, including in combinational therapy^16,17^, treatment of prostatic cancer^18–21^ and Cushing’s syndrome^22,23^. Moreover, it was shown that **Ke** could inhibit growing of several malignant and cancer cell lines^24^, inducing apoptosis through a p53 dependent pathway^25^ or inducing G_0_/G_1_ arrest by triggering mitophagy through down-regulation of COX-2 (cyclooxygenase-2)^26^.

Nowadays, due to all these diverse effects, **Ke** is regaining strong interest and new ketoconazole derivatives^27–29^ or complexes with several metal ions^30–32^ are being extensively studied. In this work, we decided to follow the pathway of modifying the existing drug and to investigate the properties of aminomethylphosphane derivatives of ketoconazole. Although a long time has passed since the first reports^33^, aminomethylphosphanes (or α-aminophosphanes) have been not considered interesting as potential drugs or components of biologically active metal complexes until the last decade. Recently, however, due to their flexibility and diversity, combined with the ease of synthesis, they started to gain a great attention. These compounds can bear an almost infinite variety of substituents with different hydrophilicities and steric demands. The potential presence of auxiliary coordinating atoms allows them to act as mono-, bi- or multidentate ligands. Our team has been working with this class of ligands and their Cu(I), Pt(II) and Ru(II) complexes and, in many cases, the properties of the derivatized molecules and their complexes are more interesting than the parent ones. For example, we worked with trisaminomethylphosphanes derived from morpholine and thiomorpholine (see for example refs^34–37^) and a variety on N4-substituted piperazines^34,35,38–41^, as well as with monoaminomethyldiphenylphosphanes^40–42^, including a derivative of a model dipeptide^43^ and the derivatives of selected fluoroquinolones: ciprofloxacin, norfloxacin^44–46^, lomefloxacin^47^ and sparfloxacin^48–50^. Some of the compounds listed above showed interesting antimicrobial and/or anti-tumour activity.

Herein we present the synthesis and characteristics of the diphenylphosphanomethyl derivative of ketoconazole and its chalcogenide derivatives (oxide, sulphide and selenide) in order to understand better the influence of a diphenylphosphanometyl(chalcogenide) moiety on the activity of the ketoconazole molecule.

## Results and Discussion

### Synthesis and characteristics of the compounds

**Figure 1:**
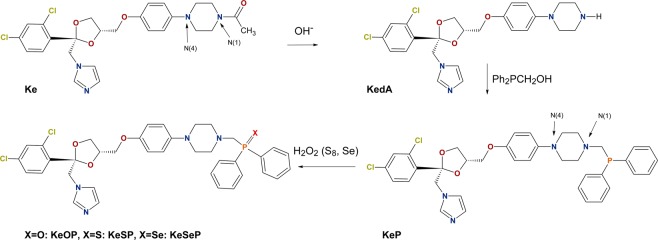
Molecular schemes of ketoconazole (**Ke**) and its derivatives.

**KeP** (Fig. 1) was synthesized from deacylated ketoconazole (**KedA**) in a modified Mannich condensation reaction of hydroxymethylphosphanes with amines^33^. Briefly, we added **KedA** to the solution of PPh_2_CH_2_OH obtained *in situ* from PPh_2_(CH_2_OH)_2_Cl by adding the excess of NEt_3_. Chalcogenides were synthesized in a reaction of **KeP** with a stoichiometric amount of H_2_O_2,_ resublimed sulphur or metallic selenium in the ultrasound bath^34,44,45,48^. To confirm the assumed structures and check the purity of the products we employed mass spectrometry and elemental analysis as well as NMR spectroscopy.

Mass spectra (Fig. S2 in ESI) confirmed the structures of the synthesized compounds. In the positive mass spectrum of the **Ke** molecule the most intense signals are [M + H]^+^ (46%) with the most intense peak at 531.2 m/z, [M + Na]^+^ (100%) at 553.1 and [2 M + Na]^+^ (63%) at 1083.5 showing strong tendency to form dimers under the experiment conditions. Phosphane **KeP** undergoes a strong fragmentation. The phosphane signal [M + Na]^+^ at 709.2 was very weak (15%) and the most intense fragmentation signals found and identified were at 489.2 [KedA + H]^+^ (6%) at 489.2 and [KedA + CH_2_]^+^ (100%) at 501.2. For the chalcogenide derivatives we observed a strongly increased stability of the molecules. In the case of **KeOP** the strongest signals were at 703.2 and 725.2 corresponding to [M + H]^+^ (6%) and [M + Na]^+^ (100%) ions respectively. A weak tendency to form dimers was reflected in the presence of the [2 M + Na]^+^ signal (5%) at 1429.4. Mass spectra of **KeSP** and **KeSeP** are very similar to one another. For both compounds we observed a small amount of the fragmentation products with dominating [KedA + CH_2_]^+^ at 501.2 with 13% or 19% intensity for **KeSP** and **KeSeP**, respectively. For **KeSP** the molecular signals were of [M + H]^+^ (29%) at 719.2 and [M + Na]^+^ (100%) at 741.2. The dimers were observed as the intense signals of [2 M + H]^+^ (51%) at 1439.4 and [2 M + Na]^+^ (32%) at 1461.4. For **KeSeP** the molecular signals were of [M + H]^+^ (48%) at 767.1 and [M + Na]^+^ (100%) at 789.1. The intensity of the signals of the dimers [2 M + H]^+^ (98%) at 1533.3 and [2 M + Na]^+^ (30%) at 1555.3 indicates that the tendency for dimer formation of the selenide derivative is the strongest among the compounds studied.

We measured the ^1^H, ^13^C{^1^H} and ^31^P{^1^H} (when applicable) NMR spectra of ketoconazole and its derivatives in CDCl_3_. Using two-dimensional COSY, HMQC and HMBC spectra we were able to assign unambiguously all the signals to the corresponding atoms in **Ke** molecule (see Figs S4–S10 and Table S1 in ESI) as well as in its derivatives (Table S2). As expected, ^1^H NMR spectrum of **Ke** is very complex and shows a set of characteristic patterns due to a presence of three different aromatic rings, racemic mixture of the chiral 2,2,4-trisubstituted 1,3-dioxolane ring and acetylated piperazine ring. It should be mentioned that the piperazine moiety in the **Ke** molecule shows four independent CH_2_ signals (carbon singlets and corresponding proton multiplets) indicating a strong binding of the acetyl group leading to the restrained rotation of the N-C bond.

Formation of **KeP** from PPh_2_(CH_2_OH) and **KedA** and further formation on the chalcogenides and the completion of the reaction is best observed by recording ^31^P{^1^H} spectra (see Tables 1, S2). In CDCl_3_ solution, PPh_2_(CH_2_OH) phosphorus atom exhibits a singlet at −10.03 ppm. Formation of the **KeP** molecule shifts this signal upfield to −27.39 ppm. Subsequent formation of chalcogenides transfers the electron density from the P atom towards a substituent, deshielding the phosphorus atom. Chemical shift for **KeOP** was 27.18, for KeSP was 35.15 and for **KeSeP** the chemical shift was 25.93 ppm with the ^1^J satellite coupling to ^77^Se equal to 721.5 Hz. All these values are typical for the diphenylphosphanomethyl ligands with N4-substituted piperazine ring attached to the P atom via methylene group and do not depend on N4 substituent^40,45^. In ^1^H and ^13^C{^1^H} NMR spectra, phosphane formation from KedA results in the new signals of methylene group being a link between **KedA** and **-PPh**_**2**_ moieties. Both proton and carbon signals are doublets with relatively small coupling constants to the phosphorus atom. This process is reflected also in the carbon signals of neighboring CH_2_ groups (position 2,6) in the piperazine ring, where a P-coupled doublet with ^3^J(P-C) around 10 Hz is observed. As for the tris(aminomethyl)phosphanes^34^ and the aminomethyldiphenylphosphanes^40,45^, formation of the chalcogenide derivatives induces dramatic changes in the signals of the groups directly bound to the P atom. This is best seen in the carbon signal of -CH_2_-P group, where formation of the P = X bond not only shifts the signal, but also significantly alters the values of ^1^J(C-P) coupling constant: from 3.6 Hz for **KeP** to 88.1, 73.6 and 66.3 Hz for **KeOP**, **KeSP** and **KeSeP**, respectively. These changes are almost repeated in the signals of the phenyl ring carbon atoms directly bound to the P atom (C(i)).

**Table 1:** Selected NMR data for ketoconazole and its derivatives (CDCl_3_).

		Ke	KeP		KeOP		KeSP		KeSeP	
^31^P{^1^H}			δ [ppm]		δ [ppm]		δ [ppm]		δ [ppm]	^1^J_SeP_ [Hz]
			−27.39		27.18		35.15		25.93	721.5
^13^C{^1^H}		δ [ppm]	δ [ppm]	^x^J_CP_ [Hz]	δ [ppm]	^x^J_CP_ [Hz]	δ [ppm]	^x^J_CP_ [Hz]	δ [ppm]	^x^J_CP_ [Hz]
-CH_2_PPh_2_	C(1) ^x=1^		61.4	3.6	58.4	88.1	63.0	73.6	63.5	66.3
	C(i) ^x=1^		138.38	12.71	132.42	97.2	131.94	77.2	130.56	69.6
	C(o) ^x=2^		132.83	18.16	131.28	9.1	131.91	10.0	132.54	10.0
	C(m) ^x=3^		128.36	6.36	128.50	11.8	128.42	11.8	128.44	11.8
	C(p) ^x=4^		128.52	S	131.86	2.7	131.64	2.7	131.74	2.7
pip	C(2;6) ^x=3^	41.41/46.31	54.55	9.08	55.54	8.2	55.24	7.3	55.15	6.4
	C(3,5)	50.63/50.89	50.46	S	50.43	s	50.40	s	50.39	s
^1^H		δ [ppm]	δ [ppm]	^x^J_CP_ [Hz]	δ [ppm]	^x^J_CP_ [Hz]	δ [ppm]	^x^J_CP_ [Hz]	δ [ppm]	^x^J_CP_ [Hz]
-CH_2_PPh_2_	H(1) ^x=2^		3.28	2.86	3.31	6.94	3.53	5.34	3.66	4.58
pip	H(2;6) m	3.76/3.61	2.83		2.81		2.69		2.67	
	H(3,5) m	3.02/3.05	3.12		3.03		2.97		2.96	

*Observed as a singlet.

**Table 2:** Activity expressed as MIC_50_ values [μM] of Ke and its derivatives against *S*. *cerevisiae* cells.

S. cerevisiae strains	Ke	KeP	KeOP	KeSP	KeSeP
WT (BY4741)	0.865	9.023	0.00416	>200	1.244
*erg6Δ*	>200	0.654	0.00482	>200	0.578
WT (W303)	6.25	12.5	3.13	>200	50
AD1-8	0.02	0.2	0.05	0.2	0.2

### Activity against yeast *Saccharomyces cerevisiae*

For the preliminary studies of the biological activity of the studied compound we chose *S*. *cerevisiae*, which is generally considered a non-pathogenic organism, but currently recognized as a potentially important agent causing opportunistic infections against immunocompromised patients^51^. In order to assess whether all the **Ke** derivatives share the mechanism of action with the parent compound, or if they might present different or additional modes of action, the inhibitory concentration MIC_50_ (Table 2) was determined on the basis of the dose-response curves (Fig. S11 in ESI) in a 48 h assay against *S*. *cerevisiae* wt strain BY4741 and the deletion mutant *erg6Δ*, which lacks the gene coding for the S-adenosylmethionine: 24-methyltransferase^52^, accumulating cholesta-5,7,24-trienol and zymosterol instead of ergosterol^53^. There are several studies reporting that the ERG6 gene deletion may increase the resistance of *S*. *cerevisiae* cells to azole compounds^54–56^. Thus, we first studied **Ke**, and confirmed this behavior, as a MIC_50_ of 0.865 μM was determined against wt cells, whereas this compound showed no activity against the mutant cells. The value determined for the wt strain is in very good agreement with previous studies carried out under similar condition (48 h, 30 °C) (e.g.^57^).

**Table 3:** MIC_50_ values [μM] for fluconazole (Flc), Ke and its diphenylphosphanomethyl derivatives towards *C*. *albicans* CAF2-1, DSY1050, B3, B4, Gu4 and Gu5.

	Flc	Ke	KeP	KeOP	KeSP	KeSeP
CAF2-1	6.25	0.05	3.13	0.78	3.13	3.13
DSY1050	0.78	0.02	0.78	0.2	0.39	0.78
B3	6.25	0.05	0.39	1.56	1.56	3.13
B4	50	0.39	3.13	3.13	3.13	6.25
Gu4	6.25	25	3.13	12.5	50	12.5
Gu5	>200	50	50	25	>200	>200

Regarding the **Ke** derivatives, they exhibit clearly distinct behavior from the parent compound, and notably, they were all active against the resistant strain to similar or even greater extent as they were against the wt strain (except **KeSP**, which was also inactive against wt cells). These results strongly suggest that the **Ke** derivatives may present different or additional modes of action underlining their antifungal activity, or override mechanism of drug resistance operative against **Ke**, since not only they are active against yeast cells that are resistant to azole compounds, and shown to be **Ke** resistant in this study, but also the behavior in relation both to the parent compound and between the two strains can change. For **KeP**, the wt cells are less sensitive (MIC_50_ 9.023 μM) than for **Ke**, while *erg6*Δ cells are very sensitive, presenting a MIC_50_ value (0.654 μM) which is even smaller than the one found for the parent compound against the wt. **KeSeP** and **KeOP** are active against both strains with similar MIC_50_ values (Table 2). Finally, as mentioned above, **KeSP** is inactive against both *S*. *cerevisiae* strains. **KeOP**, on the other hand, presents very high activity (MIC_50_ values on the nM range) against both wt and *erg6*Δ cells.

The lack of activity of **KeSP** is somewhat surprising, as it contrasts markedly with the behavior exhibited by the two other calchogenide derivatives, **KeOP** and **KeSeP**. We have, thus, hypothesized that this compound could be more efficiently exported by plasma membrane pumps. To test this hypothesis, we measured the activity of the compounds against a strain that lacks all the major ABC transporters in *S*. *cerevisiae* responsible for drug export, which is in general very susceptible to most xenobiotic compounds^58^. As control, we have also used the respective wt strain, W303. As with BY4741, W303 cells are susceptible to **Ke** and its derivatives, except for **KeSP**. This suggests that the inactivity of this compound against *S*. *cerevisiae* is a general trend, as it was observed in two different genetic backgrounds. On another hand, the MIC_50_ values are always considerably higher for W303 cells than for BY4741. This result is not unexpected, since it has been previously reported that, when grown in YPD medium, as in this study, BY4741 cells are more sensitive to Hygromycin B than W303, and they also differ in alkali-metal cation tolerance and plasma membrane potential, and in other important physiological parameters, as consequence of their different genome^59,60^. Notwithstanding, it is noteworthy that for both wt strains; the order of MIC_50_ values for the different compounds tested is almost the same, the only difference being between **KeSeP** and **KeP**, which are the second and third most active against BY4741 and in reverse order for W303 cells.

Regarding the strain lacking the major plasma membrane drug exporters, AD1-8, it is clear that all the compounds show much higher antifungal activity. The most striking result is the one for **KeSP** which displays a MIC_50_ value that is similar to **KeP** and **KeSeP**, and is more than 3 orders of magnitude lower than against the W303 cells, the wt with the same genetic bacground. The most active derivative is, again, **KeOP**. These results strongly suggest that the inactivity of **KeSP** is due to a very efficient efflux of this compound by *S*. *cerevisiae*.

### *Candida albicans* viability

#### Minimal inhibitory concentration (MIC)

Fungistatic (MIC_50_) effect of diphenylphosphanomethyl derivatives of **Ke** was evaluated in comparison to **Ke** and a triazole antifungal – fluconazole (**Flc**) (Table 3). **Flc** is the most commonly prescribed antifungal azole drug^61^ but approximately 16-fold less active *in vitro* than **Ke**^62^. **Ke** based phosphanes were less active than **Ke**; however, two (**KeP**, **KeSP**, **KeSeP**) or eight (**KeOP**) times more active than **Flc** towards *C*. *albicans* CAF2-1 strain, referential in our study.

High resistance of *C*. *albicans* towards azole based agents is related to the development of a multi-drug resistance (MDR) phenotype found among clinical *C*. *albicans* isolates, which connects with overproduction of efflux transporters located in the plasma membrane (Cdr1 and Cdr2, belonging to ATP-binding cassettes (ABCs) and Mdr1, belonging to major facilitator superfamily (MFS))^63^. In order to determine the role of MDR efflux pumps in resistance towards **Ke** derivatives, we used three pairs of *C*. *albicans* strains - CAF2-1 and DSY1050; B3 and B4; Gu4 and Gu5. The mutant with no functional *CDR1*, *CDR2* and *MDR1* genes (DSY1050) was four (**KeP**, **KeOP**, **KeSeP**) or eight (**KeSP**) times more susceptible than its parental strain (CAF2-1). *C*. *albicans* B3 and B4 strains were isolated from a patient before and after fluconazole administration, respectively. Azole-resistance of B4 results from overexpression of *MDR1*, therefore two (**KeOP**, **KeSP**, **KeSeP**) and eight (**Ke**, **KeP**) times higher resistance of B4 than B3 indicates a possible role of Mdr1p in **Ke** derivatives efflux. High levels of Mdr1p are related to the development of resistance towards voriconazole (**Vor**) and **Flc**^64–67^ but to lesser extent for other azoles, e.g. **Ke**^68–71^.

*C*. *albicans* Gu4 and Gu5 strains were also isolated from a patient before and after fluconazole administration, respectively. Contrary to B4, azole-resistance of Gu5 origins in overexpression of *CDR1* and *CDR2*. Cdr1p plays a major role in antifungal resistance in *C*. *albicans* cells by exporting most non-related xenobiotics outside fungal cells^72^. **KeSP** and **KeSeP** displayed no inhibitory effect on Gu5 strain and this strain was two and sixteen times more resistant than Gu4 strain towards **KeOP** and **KeP**, respectively. This result indicates a strong involvement of Cdr1p and Cdr2p in **Ke** derivatives resistance and that **Ke** derivatives are possibly substrates for Cdr1/Cdr2 pumps.

#### KeOP/KeP synergism with fluconazole

**Figure 2:**
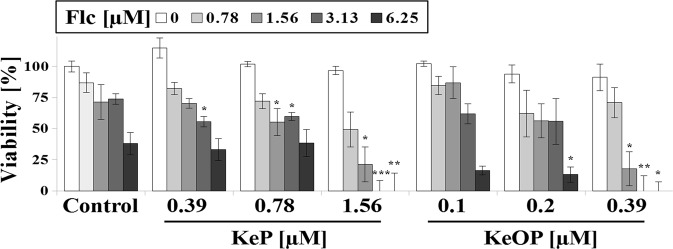
**KeP** and **KeOP** synergism with fluconazole (**Flc**). Viability [%] of *C*. *albicans* CAF2-1. Statistical analysis was performed comparing viability at corresponding **Flc** concentrations with/without **KeOP**/**KeP** (*0.01 < P < 0.05; **0.001 < P < 0.01; ***P < 0.0001). ±SD, n = 3.

In case of candidiasis treatment, a synergistic combination of clinically used azoles with other drugs is highly desirable^73,74^. One of the approaches is to inhibit activity of MDR efflux pumps by administration of competitive pump substrates among azole drugs^75^. Thus, we combined **Flc** with **KeP** or **KeOP**, since both **Ke** derivatives had most promising MIC_50_ values. **KeP** (0.39 uM) and **KeOP** (1.56 uM) reduced MIC_50_ of **Flc** in the range from 6.25 uM to 1.56 uM and when combined with higher (3.25 and 6.25 uM) **Flc** concentrations lead to fungicidal effect (Fig. 2 and Table S3 in ESI). Since azoles are fungistatic^2,76^, biocidal combination of **KeP/KeOP** with **Flc** is promising, especially, because simultaneous administration of different azoles may either lead to synergistic, subadditive or antagonistic effects^77^.

### Human adipose-derived stromal cells (hACSs) studies

#### Half maximal inhibitory concentration (IC_50_) assessment

**Figure 3:**
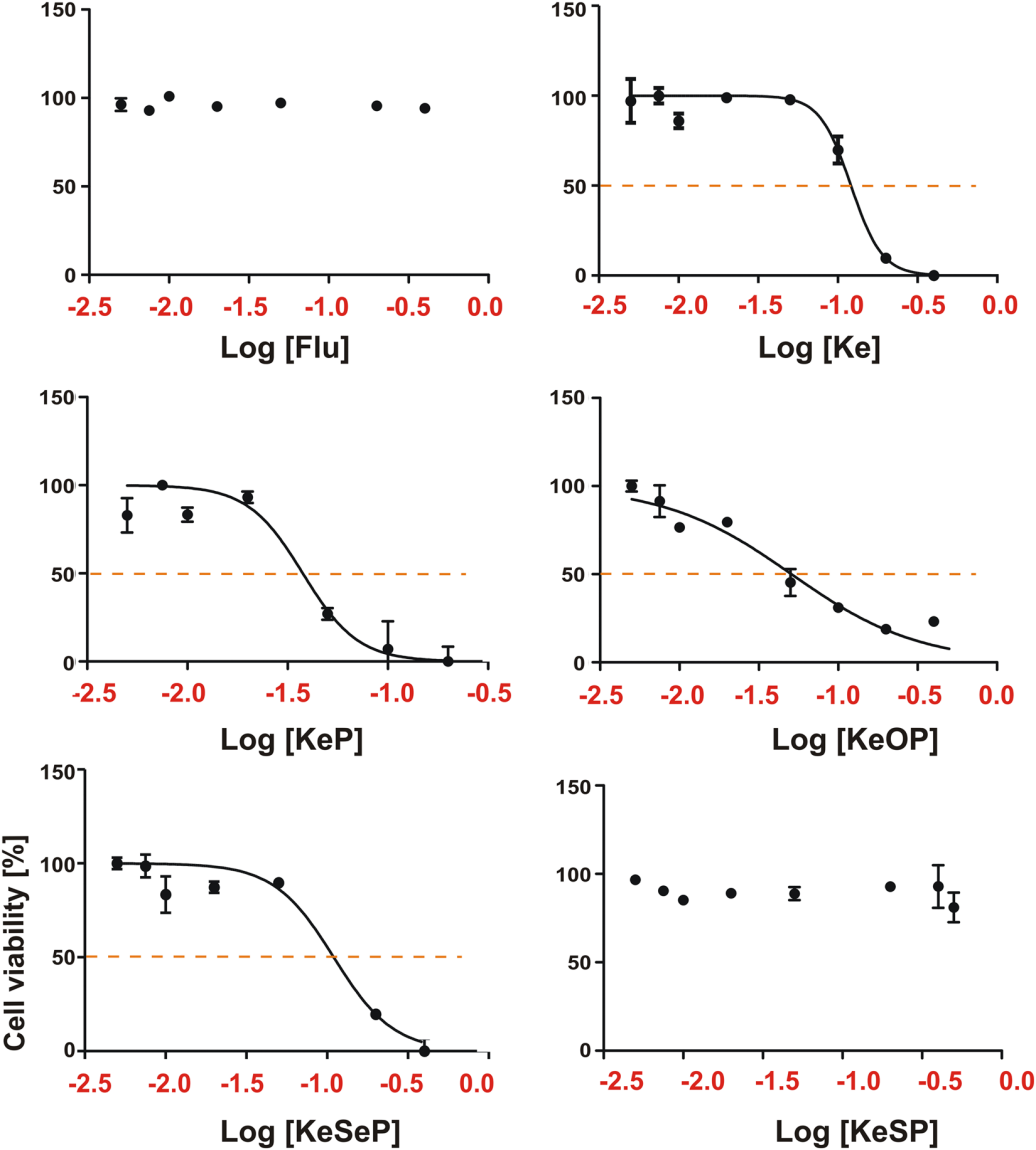
hACSs viability in the presence of different azole compounds. Percentage of cell survival is plotted against the logarithm of treatment concentrations. Calculated IC_50_ values are: 120.8 for **Ke**, 37.65 for **KeP**, 50.76 for **KeOP** and 109.8 μM for **KeSeP**. It was not possible to determine the IC_50_ values for **Flc** and **KeSP**.

In order to assess the activity of the ketoconazole and its derivatives against human cells, IC_50_ values for **Ke**, **KeP**, **KeOP** as well as **KeSeP** for hASCs (human adipose-derived stromal cells) were obtained from the dose-response curves that were prepared using TOX-8 Assay after a 24-hours stimulation (Fig. 3).

The cytotoxicity assay indicated **KeP** (IC_50_ = 37.65 µM) as the most active compound. The cytotoxicity of **KeOP** (IC_50_ = 120.8 µM) and **KeSeP** (IC_50_ = 109.8 µM) were significantly lower, and the lowest cytotoxic effect was found for **Flc** and **KeSP** (Fig. 3), which were not cytotoxic in the tested concentration range. These data stand in good agreement with other results, that show a relatively small cytotoxicity of **Flc**^78^. In this study, the IC_50_ value for **Ke** is 120.8 µM. However, contradictory data regarding cytotoxicity of **Ke** has been reported^78^, which might be due to the use of different cell sources. It was previously shown by Benkoő *et al*. that the IC_50_ value of **Ke** for whole population of human bone marrow cells was equal to 6,27 mg/L (11.8 µM). Moreover, the IC_50_ of **Ke** was almost 3-fold higher for murine bone marrow cells in comparison to the human cells^78^. In turn, Haegler *et al*. showed that in HepG2 cells (liver hepatocellular carcinoma cell line), **Ke** cytotoxic effects started to be noticed at 50 µM. In contrast, in HepaRG cells, cytotoxicity started at 100 µM^79^.

#### Apoptosis level of hASCs after 24-hours stimulation with **Ke**, **KeP** and **KeOP** (20 µM)

**Figure 4:**
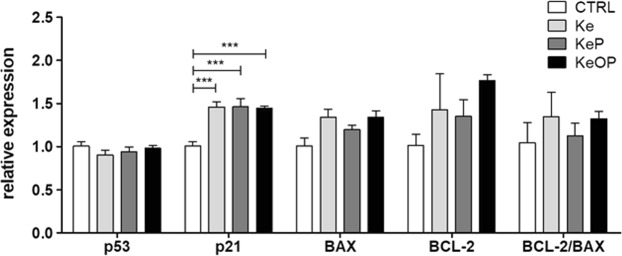
Apoptosis level of hACSs treated with 20 µM **Ke**, **KeP** and **KeOP** for 24 hours. Results expressed as mean ± SD. Statistical significance indicated as three asterisks ***p < 0.001.

Apoptosis is characterized by cell shrinkage, alternations in the cell membrane and mitochondria, nuclear condensation, as well as DNA fragmentation and protein degradation by caspases^80^. It was shown that BCL-2 transcript protects cells from apoptosis by associating with mitochondria and inhibiting their release of cytochrome c, whereas BAX promotes apoptosis. Moreover, p53 gene expression is associated with induction of apoptosis^80,81^. Interestingly, p21 transcript plays a dual role in cell death and survival, i.e., it inhibits apoptosis through induction of cell cycle arrest and DNA repair, but p21-arrested cells may undergo apoptosis following activation of other pro-apoptotic genes^81^. In this study, we observed significant (p < 0.001) elevated expression of p21 transcript in cells challenged with **Ke**, **KeP** or **KeOP** when compared to the control group. Moreover, the relative expression levels of both BAX and BCL-2 were higher in cells treated with **Ke**, **KeP** or **KeOP**, but the differences were not significant. Interestingly, none of the compounds increased the expression of a master regulator of apoptosis, which is a p53 transcript (Fig. 4). Furthermore, no significant changes in the expression of p53 gene were observed when compared to control cells. This observation might suggest that the tested dosage of **Ke**, **KeP** and **KeOP**, which was 20 µM, did not induce apoptosis in hASC cultures. which might be a positive feature light for their future potential clinical application in antifungal therapy.

#### Mitochondria network and F-actin in hASCs

**Figure 5:**
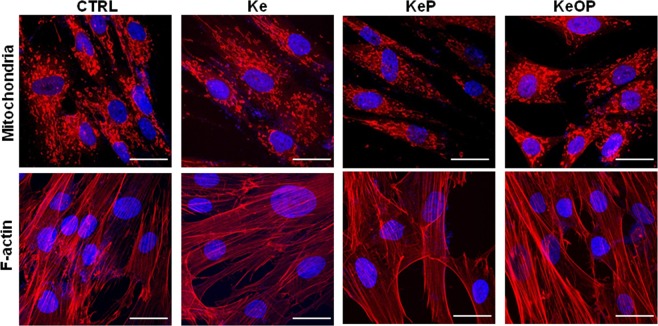
Visualization of mitochondrial network and F-actin of hASCs treated with **Ke**, **KeP** and **KeOP** (20 µM; 24 h) using MitoRed and Phalloidin Atto 590 staining, respectively. Scale bar: 50 µm.

Cell morphology was evaluated using MitoRed and Phalloidin Atto 590 staining after 24 h stimulation with **Ke**, **KeP** and **KeOP** (20 µM). MitoRed is a vital dye which interacts with functional mitochondria due to their high membrane mitochondrial potential. Decreased fluorescence of MitoRed stain has been associated with reduced mitochondrial membrane potential^82^. Patalano *et al*. showed that **Ke** treatment induces depolarization of mitochondria which leads to the activation of apoptosis in mouse corticotroph tumour cell line (AtT20-D16)^83^. Moreover, Haegler *et al*. showed that **Ke** decreases the mitochondrial potential in HepG2 cells, which was associated with decreased mitochondrial DNA synthesis and accumulation of mitochondrial superoxide that induce apoptosis^84^. In this work, the mitochondrial staining indicates the lowest mitochondrial membrane potential in **KeP** treated cells, as well as the lowest number of mitochondria. (Fig. 5). This might suggest a reduced antioxidative capacity. In turn, phalloidin staining showed a multilayer growth pattern in cells treated with **Ke** and **KeOP**. These cells where characterized by close contact with a relatively dense growth pattern, while hASCs treated with **KeP** were characterized by a loose growth pattern. It can be concluded that the compounds tested, i.e. **Ke**, **KeP** and **KeOP**, affect in different ways the morphological features of progenitor cells. Moreover, **KeP** seems to affect negatively cellular morphology and mitochondrial activity.

### Docking to CYP51 of *C*. *albicans*

As it has been said in the introduction, primary molecular target of **Ke** is cytochrome P450 14-alpha- demethylase. Mechanism of action is pretty simple. **Ke**, like other azoles, acts as an competitive inhibitor binding cytochrome Fe atom by N atom from imidazole ring and therefore blocking the enzyme active site. It was proven by several X-ray structures of the enzyme with different azoles. For **Ke** there one can find a structure of human CYP51 inhibited by the **Ke** molecule^13,14^, and only recently a structure of fungal CYP51 from *C*. *albicans* in complex with posaconazole was determined^15^. To check if the presented here derivatives can also target this enzyme we performed Molecular Docking using AutoDock Vina software^85^.

**Figure 6:**
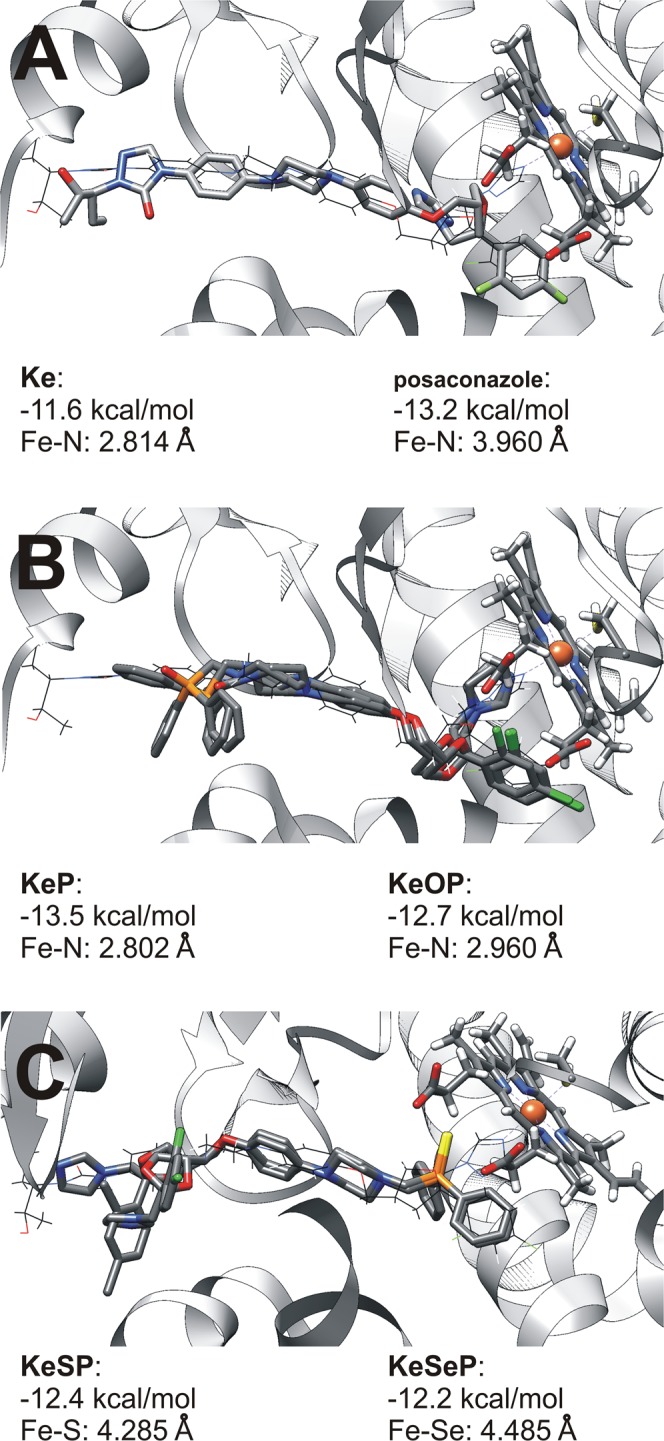
Docking results: heme of the *C*. *albicans* CYP51 and the best-fit docked compounds; posaconazole position from the original crystal structure^15^ is also indicated (as a black wire). (**A**) docked posaconazole, (**B**) **Ke**, **KeP**, **KeOP**, C: **KeSP**, **KeSeP**.

The docking results together with the best-fit structures are summarized in Fig. 6. The reference structure is the CYP51-posaconazole complex and the location of posaconazole serves to determine the correct binding mode. Posaconazole itself was also subjected to the docking to validate the docking procedure. Interestingly, while the correct overall binding mode is found for posaconazole, the triazole ring is rotated so that the Fe-N distance is much larger than in the experimental crystal structure (3.96 Å vs. 2.08 Å). However, the calculated binding affinity for posaconazole is the second strongest among the molecules studied, next only to **KeP**. For all five **Ke** derivatives, the proper location was found by the docking algorithm, which is a good indication of their binding affinity and the proper shape of the binding site cavity. The search space was large enough (cubic box, a = 80 Å) not to force the ligand into the cavity. The best-fit structures are always located in the binding site and oriented properly (that is, the heme iron atom is coordinated by the N atom in the imidazole ring of a given derivative), with the two important exceptions of **KeSP** and **KeSeP**, for which the sulfur or selenium atoms are oriented towards the heme iron. The resulting structure is “head to tail” with respect to posaconazole and the remaining **Ke** derivatives. This might arise from the stronger preference of the Fe-S(Se) interaction over the Fe-N contact, or from an increased volume of the P-S(Se) fragment. The matter of possible Fe-S(Se) coordination will be further investigated experimentally; nevertheless, both **KeSP** and **KeSeP** are also located in the correct cavity. Taking into account the approximate nature of the docking score function, it is not possible to determine whether the investigated **Ke** derivatives bind more or less strongly to the protein than **Ke** itself, but no dramatic changes were noted, suggesting that binding affinities among the analyzed **Ke** derivatives should be of similar order.

For **KeP** and **KeOP**, the ligands oriented as **Ke**, an analysis of the vicinity of the phosphorus atom was carried out. For **KeP**, the P atom interacts via its lone pair with the Pro230 ring (the closest distance P-C(Pro230) is 3.811 Å). Moreover, the P atom is also in potential contact with the phenyl ring of Phe233 (the closest P-C(Phe233) distance is 4.766 Å). In the case of **KeOP**, the direct environment around the ligand is somewhat different: Pro230, Phe233, Phe235 and Phe380 form a hydrophobic cage surrounding the –PPh_2_ phenyl rings of the ligand, while the P = O moiety is in contact with the Tyr64 phenol group (the closest (**KeOP**)O…C(Tyr64) distance is 3.313 Å, and the O…O(phenol,Tyr64) distance is 4.667 Å). These values must be taken as a rough estimation, due to the aforementioned nature of the docking scoring function, but they represent a possible diversity of bonding between the protein and the phosphane moiety of the **Ke** derivatives. In particular, the possibility of additional stabilization by interactions with a hydrophobic cage could add to the binding strength of **KeP**.

## Conclusions

Derivatization of ketoconale with diphenylphosphanomethyl moiety gave an opportunity to develop a set of similar molecules differing only by the direct environment of the phosphorus atom. Four new derivatives of ketoconazole (**Ke**) were synthesized: diphenylphosphane (**KeP**) and its chalcogenides: oxide (**KeOP**), sulphide (**KeSP**) and selenide (**KeSeP**). Their structures were analyzed using NMR spectroscopy and mass spectrometry.

The derivatives of ketoconazole synthesized and tested in this work, especially **KeP** and **KeOP**, proved to be promising antifungal compounds. A strong antifungal activity, in some occasions surpassing largely the parent compound, was shown for selected strains of *S*. *cerevisiae* and also in the detailed study for *C*. *albicans*, especially in synergy with fluconazole. All compounds tested are substrates for pumps expelling drugs (mainly Cdr1 and Cdr2) from *C*. *albicans* cells, and simultaneous administration of fluconazole allowed a significant reduction in concentrations of ketoconazole derivatives tested and low survival of the fungus. Simulations of docking of the derivatives to the cytochrome P450 14-α-demethylase (the azoles’ primary molecular target) did not show large differences between their binding strength, also in comparison with ketoconazole, proving that they are capable of inhibiting this enzyme. While **KeP** and **KeOP**, like **Ke**, form a bond between nitrogen atom from the azole ring and the heme iron atom, for **KeSP** and **KeSeP**, a reversed placement in the hydrophobic cage was the preferred one. This opens a possibility of a different/secondary action mechanism for these compounds and will be studied in detail.

In what concerns cytotoxicity towards hACSs, the individual compounds had displayed some activity, except for **KeSP** which was not toxic. Two ketoconazole derivatives were further studied (**KeP** and **KeOP**) and in both cases we observed an increased level of the p21 gene transcript, but no changes in the level of p53 transcript. A decreased mitochondrial membrane potential was also apparent. This may indicate that all the new ketoconazole derivatives have a similar mechanism of action and block the lanosterol 14α-demethylase and thus inhibit the production of ergosterol in *C*. *albicans* membranes. The IC_50_ value in the cytotoxicity assay with human cells was significantly higher than the MIC_50_ for *C*. *albicans*, especially when the compounds were used with fluconazole. Therefore, we conclude that phosphane derivatives of ketoconazole are a promising group of compounds with potential use in the treatment of mycoses.

Taking into account all our results, we conclude that **Ke** based phosphane is a very interesting ligand and enables a significant expansion of possible metal complexes bearing the **Ke** moiety. Currently, we are working on copper(I) and ruthenium(II) complexes with **KeP**. Moreover, we are going to study phosphanes bearing more than one **Ke** moieties.

## Experimental Part

### Syntheses

Reagents and solvents used for synthesis were purchased from Sigma-Aldrich, Poland and used without further purifications. All syntheses were performed under inert (N_2_) atmosphere using standard Schlenk techniques. Deacylated ketoconazole (**KedA**; 1-4-[2-(2,4-Dichlorophenyl)-2-imidazol-1-ylmethyl-[1,3]-dioxolan-4-ylmethoxy]phenyl-piperazine; M = 489.4 g/mol) and PPh_2_(CH_2_OH)_2_Cl (M = 282.7 g/mol) were prepared following literature procedures^86,87^.

#### Ph_2_PCH_2_-Ke (**KeP**)

Triethylamine (3 mL) was added drop wise to a solution containing 0.3207 g of PPh_2_(CH_2_OH)_2_Cl (1.134 mmol) in 10 mL of MeOH in an ice bath. After stirring for 30 min, an MeOH solution (15 mL) of **KedA** (0.5550 g; 1.134 mmol) was added. The mixture was stirred for 1 h at room temperature and its volume was reduced by 1/3 of initial volume under a stream of nitrogen. Then 100 mL of water was slowly added. The obtained white solid was filtered, washed five times with water and dried under reduced pressure for 24 h. Yield 61%. Anal. Calc. for C_37_H_37_Cl_2_N_4_O_3_P (M = 687.6 g/mol): C, 64.63; H, 5.42; N, 8.15. Found: C, 64.34; H, 5.45; N, 8.11%. MS ESI (+):489.2 [KedA + H]^+^ (6%), 501.2 [KedA + CH_2_]^+^ (100%), 709.2 [M + Na]^+^ (15%).

#### Ph_2_P(O)CH_2_-Ke (**KeOP**)

**KeOP** was obtained in a dichloromethane-acetonitrile mixture (1:1) (10 mL) through the reaction of **KeP** (0.1081 g; 0.157 mmol) placed in an ice bath with an equimolar amount of H_2_O_2_ (30% solution in water: 16 μL; 0.157 mmol). After stirring for 30 min, the solvent was evaporated under vacuum. The remaining white solid was washed twice with water and dried for 24 h under vacuum to remove any occluded solvent molecules. Yield 94%. Anal. Calc. for C_37_H_37_Cl_2_N_4_O_3_P (M = 703.6 g/mol): C, 62.79; H, 5.33; N, 7.92. Found: C, 62.83; H, 5.33; N, 7.92%. MS ESI (+):703.2 [M + H]^+^ (6%), 725.2 [M + Na]^+^ (100%), [2 M + Na]^+^ 1429.4 (5%).

#### Ph_2_P(S)CH_2_-Ke (**KeSP**)

To **KeP** (0.0983 g; 0.143 mmol) in chloroform (15 mL) an equimolar amount of resublimed sulfur (0.00457 g; 0.143 mmol) was added. The mixture was placed in an ultrasonic bath for 1 h at room temperature. The yellowish solid obtained after removing the solvent was redissolved in a minimal volume of methanol and precipitated with large amounts of water. The solid obtained was filtered and dried for 24 h under vacuum. Yield 68%. Anal. Calc. for C_37_H_37_Cl_2_N_4_O_3_PS (M = 719.7 g/mol): C, 61.75; H, 5.18; N, 7.78. Found: C, 61.40; H, 5.22; N, 7.74%. MS ESI (+): 501.2 [KedA + CH_2_]^+^ (13%), 719.2 [M + H]^+^ (29%), 741.2 [M + Na]^+^ (100%) 1439.4 [2 M + H]^+^ (51%), 1461.4 [2 M + Na]^+^ (32%).

#### Ph_2_P(Se)CH_2_-Ke (**KeSeP**)

An equimolar mixture of **KeP** (0.1026 g; 0.149 mmol) and metallic selenium (0.01178 g; 0.149 mmol) in chloroform (15 mL) was placed in an ultrasonic bath until the traces of solid Se disappeared (~1 h). The yellowish solid obtained after removing the solvent was redissolved in a minimal volume of methanol and precipitated with a large amount of water. The solid obtained was filtered and dried for 24 h under vacuum. Yield 73%. Anal. Calc. for C_37_H_37_Cl_2_N_4_O_3_PSe (M = 766.6 g/mol): C, 57.97; H, 4.87; N, 7.31. Found: C, 57.66; H, 4.90; N, 7.31%. MS ESI (+):501.2 [KedA + CH_2_]^+^ (19%), 767.1 [M + H]^+^ (48%), 789.1 [M + Na]^+^ (100%), 1533.3 [2 M + H]^+^ (98%), 1555.3 [2 M + Na]^+^ (30%).

### Methods

Elemental analyses were performed on a Vario EL3 CHN analyzer for C, H, and N, and they were within ±0.3% of the theoretical values. NMR spectra were recorded on a Bruker AMX 500 spectrometer (at 298 K) with traces of solvent as an internal reference for ^1^H (CDCl_3_: 7.27 ppm) and ^13^C spectra (CDCl_3_: 77.0 ppm) and 85% H_3_PO_4_ in H_2_O as an external standard for ^31^P. All the NMR data are presented in ESI (Tables S1, S2) Mass spectra were recorded on a Bruker Daltonics micrOTOF-Q mass spectrometer equipped with an electrospray ionization (ESI) source and operated in positive ion mode.

### Saccharomyces cerevisiae

The following strains of *S*. *cerevisiae* were used in this study: BY4741 (wild type towards *erg6Δ*, ACC Y00000; MATa; *his3Δ1; leu2Δ0; met15Δ0; ura3Δ0*), *erg6Δ* (ACC Y00568; BY4741 isogenic with *YML008c::kanMX4*), W303 (wild type towards AD1-8; MAT*α*; *leu2-3*,*112 trp1-1 can1-100 ura3-1 ade2-1 his3-11*,*15* AD1-8 (W303 isogenic with *pdr1-3*, *ura3*, *his1*, *yor1∆::hisG*, *snq2∆::hisG*, *pdr5∆::hisG*, *pdr10∆::hisG*, *pdr11∆:: hisG*, *ycf1∆::hisG*, *pdr3∆::hisG*, *pdr15∆::hisG*). BY4741 and *erg6Δ* were acquired from EUROSCARF, Frankfurt, Germany; W303 and AD1-8 were kind gifts from Prof. A. Goffeau, Chaire Internationale Blaise Pascal, Lab. Genet. Mol., École Normale Supérieure, Paris, and Prof. M. Ghislain, Université Catholique de Louvain, Faculté des sciences Agronomiques, Louvain-la-Neuve, Belgium. Visible turbid overnight cultures of *S*. *cerevisiae* strains were prepared and diluted to A_600_ ≈ 0.1 in fresh Yeast Extract – Peptone - Dextrose (YPD) medium. The cultures were transferred to 96 microtiter well plates containing aliquots of serially diluted antifungal drugs (**Ke** and its derivatives: **KeP**, **KeOP**, **KeSP**, **KeSeP**), prepared from DMS stock solutions and drug-free controls. The yeast cultures were statically incubated at 30 °C for 48 hours with compounds^88^. The dose-response curves were analysed with GraphPad Prism 5 software (San Diego, USA) or a microplate reader ASYS UVM (Biogenet). All values presented are the mean of at least three independent experiments.

### Candida albicans

#### *C*. *albicans* strains and growth conditions

The *C*. *albicans* CAF2-1 (Genotype/Description: *ura3Δ::imm434/URA3*^89^) and DSY1050 (Genotype/Description: *cdr1Δ::hisG/cdr1Δ::hisG*/*cdr2Δ::hisG/cdr2Δ::hisG*/*mdr1Δ::hisG-URA3-hisG/mdr1Δ::hisG*^90^) strains used in this study were generous gift from D. Sanglard (Lausanne, Switzerland)^91,92^ whereas *C*. *albicans* B3 and Gu4 (Genotype/Description: clinical isolates fluconazole sensitive)^93^), B4 (Genotype/Description: Clinical isolate fluconazole resistant due to the overexpression of *MDR1*^93^) and Gu5 (Genotype/Description: Clinical isolate fluconazole resistant due to the overexpression of *CDR1* and *CDR2*^93^) were a generous gift from prof. S. Milewski (Gdańsk, Poland)^94^ and prof. J. Morschhäuser (Wurzburg, Germany)^93^. All strains were grown at 37 °C on Sabouraud medium (Biomerieux).

#### Minimal inhibitory concentration (MIC) determination

The antifungal activity was determined on the 96-well polystyrene microtiter plates (Sarstedt) according to Clinical and Laboratory Standards Institute M27-A3^95^. The different strains were incubated with antifungal compounds (fluconazole (**Flc**) (Sigma-Aldrich), **Ke** and **Ke** derivatives) in the range of concentrations 0.01–200 μM. The compounds were dissolved in DMSO (10 mM) and then serially diluted in RPMI-1640 (ThermoFisher) medium to obtain the final concentration range. Inocula of all strains were prepared as suspensions with turbidity of 0.5 McFarland standard in sterile 0.85% saline and diluted in RPMI-1640. After inoculation, each well contained appr. 5 × 10^2^ CFU/mL. The plates were cultivated for 48 h at 37 °C. Optical density was measured at A_490nm_ using a microplate reader ASYS UVM 340 (Biogenet). The concentrations which resulted in ≥50% growth inhibition were determined as MIC_50_.

#### KeOP/KeP synergism with fluconazole

The synergistic effects of **KeOP**/**KeP** with **Flc** were determined using the M27-A3 method with modifications. **Flc** and **KeOP**/**KeP** stock solutions in DMSO (10 mM) were (alone and simultaneously) diluted in RPMI-1640 medium to obtain final concentrations of^1/8^; ^1/4^; ^1/2^; 1 × MIC_50_ towards *C*. *albicans* CAF2-1 strain in microdilution sterile plates (Sarstedt). Statistical significance analysis was performed using Student’s *t*-test (binomial, unpaired) comparing viability in the presence of corresponding **Flc** concentrations with/without **KeOP**/**KeP**.

### Human Adipose Tissue-derived Stem Cells (hASCs)

Unless indicated otherwise, all reagents used in this study were purchased from Sigma-Aldrich (Poland).

#### Cells isolation and culture

Human Adipose Tissue-derived Stem Cells (hASCs) were isolated from subcutaneous adipose tissue fragments harvested from non-diabetic female donors (age range 33–38, n = 2) during standard surgical procedure of total hip arthroplasty. Immediately after collection of tissue, the samples were placed in sterile tissue-transport medium (Hank’s Balanced Salt Solution (HBSS) supplemented with 1% Penicillin/Streptomycin/Amphotericin B solution (P/S/A). Mesenchymal stromal cell isolation was performed using enzymatic digestion of extracellular matrix with collagenase type I. Before digestion, tissue specimens were washed twice with HBSS and incubated in collagenase type I solution (1 mg/mL) at 37 °C for 40 min. The digest was subsequently centrifuged (1200 × *g*; 10 minutes; room temperature) and the cell pellet was resuspended in Dulbecco’s modified Eagle’s medium (DMEM) with Nutrient F-12 Ham, 10% of Fetal Bovine Serum (FBS), and 1% of P/S/A, transferred to a culture flask, and kept in culture under optimal conditions (37 °C in a humidified atmosphere of 5% CO_2_). The medium was refreshed every 2–3 days. At 80–90% confluence, the cells were passaged using trypsin solution (TrypLE Express, Life Technologies, California, USA). At passage 3, hASCs were collected and cellular phenotype was confirmed by the high expression of markers CD44, CD29, CD73, CD105 and low expression of CD45. Moreover, the trilineage differentiation capacity of ASCs was confirmed as previously described^96^.

#### Half maximal inhibitory concentration (IC_50_) determination

The IC_50_ values were determined using *in vitro* Toxicology TOX-8 Assay Kit (resazurin based) according to the manufacturer’s protocol. For this assay, cells were seeded in a 96-well plate at a density of 5 × 10^3^ cells/well. The cells were pre-incubated in drug-free complete medium for 24 h before adding various concentrations of the compounds to be investigated. Stock solutions of the compounds were prepared in DMSO following serial dilutions in DMEM with Nutrient F-12 Ham complete medium. The cells exposure period was 24 h. After that time media were replaced with 10% v/v resazurin dye solution in fresh complete culture medium. Incubation was carried out for 2 h at 37 °C in the CO_2_ incubator. Reduction of the dye was measured spectrophotometrically at 600 nm and 690 nm reference wavelength (Epoch, Biotek). Absorbance measurements allowed the determination of viable treated cells relative to untreated controls using the inflection point of a dose-response graph. IC_50_ values were estimated as duplicates of triplicate readings in two independent experiments.

#### Evaluation of cellular apoptosis

Cellular apoptosis level was assessed using quantitative reverse transcriptase real-time polymerase chain reaction (qRT-PCR). First, the cells were seeded in a 24-well plate at a concentration of 3 × 10^4^ cells/well in DMEM with Nutrient F-12 Ham complete medium and left in the CO_2_ incubator overnight to allow cells to attach. The compounds (**Ke**, **KeP** and **KeOP**) were diluted in the fresh complete medium to a final concentration of 20 μM. After 24 h of challenge with the compounds, the cells were homogenized with TRI Reagent and total RNA was isolated by phenol-chloroform extraction and ethanol precipitation^97^. RNA purity and quantity were measured at a wavelength of 260 and 280 nm (Epoch, Biotek). Genomic DNA was removed with DNase I, RNase-free (1 U/L) (Thermo Scientific, Poland) and cDNA was synthesized using RevertAid First Strand cDNA Synthesis Kit (Thermo Scientific, Poland). 150 ng of total RNA served as a template for a single reaction using T100 Thermo Cycler (Bio-Rad, USA). The expression levels of p53 tumor suppressor, cyclin dependent kinase inhibitor 1A (p21), apoptosis regulators BAX and BCL-2 were determined. qRT-PCR was performed on a CFX Connect^TM^ Real-Time PCR Detection System (Bio-Rad) using SensiFast SYBR & Fluorescein Kit (Bioline, Cincinnati, OH, USA) and primer pairs as listed in Table S4 in ESI. 500 nM of specific primers and 1 µL of cDNA in a 10 µL final volume were applied in each reaction. The quantitative expression of the genes was calculated by the 2^−ΔΔCT^ method using GAPDH as housekeeping genes.

Statistical analysis was performed with GraphPad Prism 5 software (San Diego, USA). All data values are presented as mean ± SD, measured in triplicates or more. Statistical significance between groups was determined using two-way ANOVA grouped analysis.

#### Confocal microscopy imaging

The mitochondrial network was visualized using MitoRed staining. After 24-hours treatment with either **Ke**, **KeP** or **KeOP**, the media were removed and replaced with MitoRed dye in 1:1000 dilution in fresh complete media. Cells were incubated for 30 minutes at 37 °C, then washed with Phosphate Buffered Saline (PBS), fixed with 4% paraformaldehyde (PFA) solution, washed three times and nuclei were counterstained with diamidino-2-phenylindole (DAPI).

In order to perform F-actin imaging, cells were fixed with 4% PFA solution, washed with PBS and then permeabilized using 0.5% TX-100 (incubation 15 minutes, room temperature). The cells were rinsed and incubated with Phalloidin-Atto 590 solution in PBS (dilution 1:1000) at 37 °C for 30 minutes in the dark. After washing, the cells were counterstained with DAPI.

Cells were observed and images were taken using confocal microscopy performed with a Leica TCS-SP8 laser scanning confocal microscope. Data were further processed with the use of ImageJ software.

### Docking studies

Docking calculations were performed using the X-ray structure of *C*. *albicans* CYP51 in complex with posaconazole^15^ (PDB code 5FSA) as the macromolecular host, and the set of ketoconazole derivatives as the ligands. Posaconazole was also included in the set as a validation test. The DFT-optimized structures (the Gaussian 16, Rev. B.01 program^98^ with ωB97XD functional^99^ and the 6-311 + G(d,p) basis set) served as initial ligand structures. The macromolecule consisted of the protein chain A, together with the heme cofactor, but without posaconazole. The docking of the ligands was carried out using a cubic box with an 80 Å edge, centered on the iron atom of the heme cofactor, as the search space; up to nine structures for each search were retained for further analysis. For two of the ligands (**KeSP**, **KeSeP**), docking runs were also repeated with a smaller box (a = 40 Å). A docking run for each ligand was replicated three times with different random seeds, and generally the results were consistent between the replicas. Preprocessing of the structures and visualization of the results was carried out with AutoDockTools program of the MGLTools package^100^ and UCSF Chimera 1.11.2_b41376 graphic program^101^, while the docking itself was performed with the AutoDock Vina software ver. 1.1.2^85^.

## Supplementary information

Supplementary material
