# Supplementary material for: New diphenylphosphane derivatives of ketoconazole are promising antifungal agents
Source: Sci Rep. 2019 Nov 7;9:16214. doi: 10.1038/s41598-019-52525-7 (PMC6838151; doi:10.1038/s41598-019-52525-7)
Supplement: Supplementary file 1 — Supplementary material [file 41598_2019_52525_MOESM1_ESM.pdf]

Supplementary Materials

New diphenylphosphane derivatives of ketoconazole are promising antifungal agents.

Rodrigo F. M. de Almeida,[a] *Filipa C. Santos,*[a]
Krzysztof Marycz,[b] *Michalina Alicka,*[b] *Anna Krasowska,*[c] *Jakub Suchodolski,*[c]
Jarosław J. Panek,[d] *Aneta Jezierska*[d] *and Radosław Starosta*[a,d,]*

Abstract

Four new derivatives of ketoconazole (**Ke**) were synthesized: diphenylphosphane (**KeP**), and phosphane chalcogenides: oxide (**KeOP**), sulphide (**KeSP**) and selenide (**KeSeP**). These compounds proved to be promising antifungal compounds towards *Saccharomyces cerevisiae* and *Candida albicans*, especially in synergy with fluconazole. Simulations of docking to the cytochrome P450 14α-demethylase (azoles' primary molecular target) proved that the new **Ke** derivatives are capable of inhibiting this enzyme by binding to the active site. Cytotoxicity towards hACSs (human adipose-derived stromal cells) of the individual compounds was studied and the IC_{50} values were higher than the MIC_{50} for *C. albicans* and *S. cerevisiae*. **KeP** and **KeOP** increased the level of the p21 gene transcript but did not change the level of p53 gene transcript, a major regulator of apoptosis, and decreased the mitochondrial membrane potential. Taken together, the results advocate that the new ketoconazole derivatives have a similar mechanism of action and block the lanosterol 14α-demethylase and thus inhibit the production of ergosterol in *C. albicans* membranes.

Keywords: ketoconazole; phosphanes; antifungal activity; *Saccharomyces cerevisiae*; *Candida albicans*; cytotoxicity; human adipose-derived stromal cells; molecular docking; ergosterol

[a] Centro de Química e Bioquímica, Departamento de Química e Bioquímica, Faculdade de Ciências da Universidade de Lisboa, Campo Grande, 1749-016 Lisboa, Portugal
[b] Department of Experimental Biology, Faculty of Biology and Animal Science, Wroclaw University of Environmental and Life Sciences, Norwida 27B, 50-375 Wroclaw, Poland
[c] Faculty of Biotechnology, University of Wroclaw, F. Joliot-Curie 14a, 50-383 Wroclaw, Poland
[d] Faculty of Chemistry, University of Wroclaw, F. Joliot-Curie 14, 50-383 Wroclaw, Poland
*- corresponding author: radoslaw.starosta@chem.uni.wroc.pl

Ke: CC(=O)N1CCN(CC1)c2ccc(cc2)OC[C@H]4CO[C@@](Cn3cncc3)(O4)c5ccc(Cl)cc5Cl

KedA: Clc1ccc(c(Cl)c1)[C@@]5(Cn2cncc2)OC[C@H](COc3ccc(cc3)N4CCNCC4)O5

KeP: Clc1ccc(c(Cl)c1)[C@@]7(Cn2cncc2)OC[C@H](COc3ccc(cc3)N6CCN(CP(c4ccccc4)c5ccccc5)CC6)O7

KeOP: O=P(CN1CCN(CC1)c2ccc(cc2)OC[C@H]4CO[C@@](Cn3cncc3)(O4)c5ccc(Cl)cc5Cl)(c6ccccc6)c7ccccc7

KeSP: S=P(CN1CCN(CC1)c2ccc(cc2)OC[C@H]4CO[C@@](Cn3cncc3)(O4)c5ccc(Cl)cc5Cl)(c6ccccc6)c7ccccc7

KeSeP: Se=P(CN1CCN(CC1)c2ccc(cc2)OC[C@H]4CO[C@@](Cn3cncc3)(O4)c5ccc(Cl)cc5Cl)(c6ccccc6)c7ccccc7

Fig.S1 Smiles notations for the studied compounds.

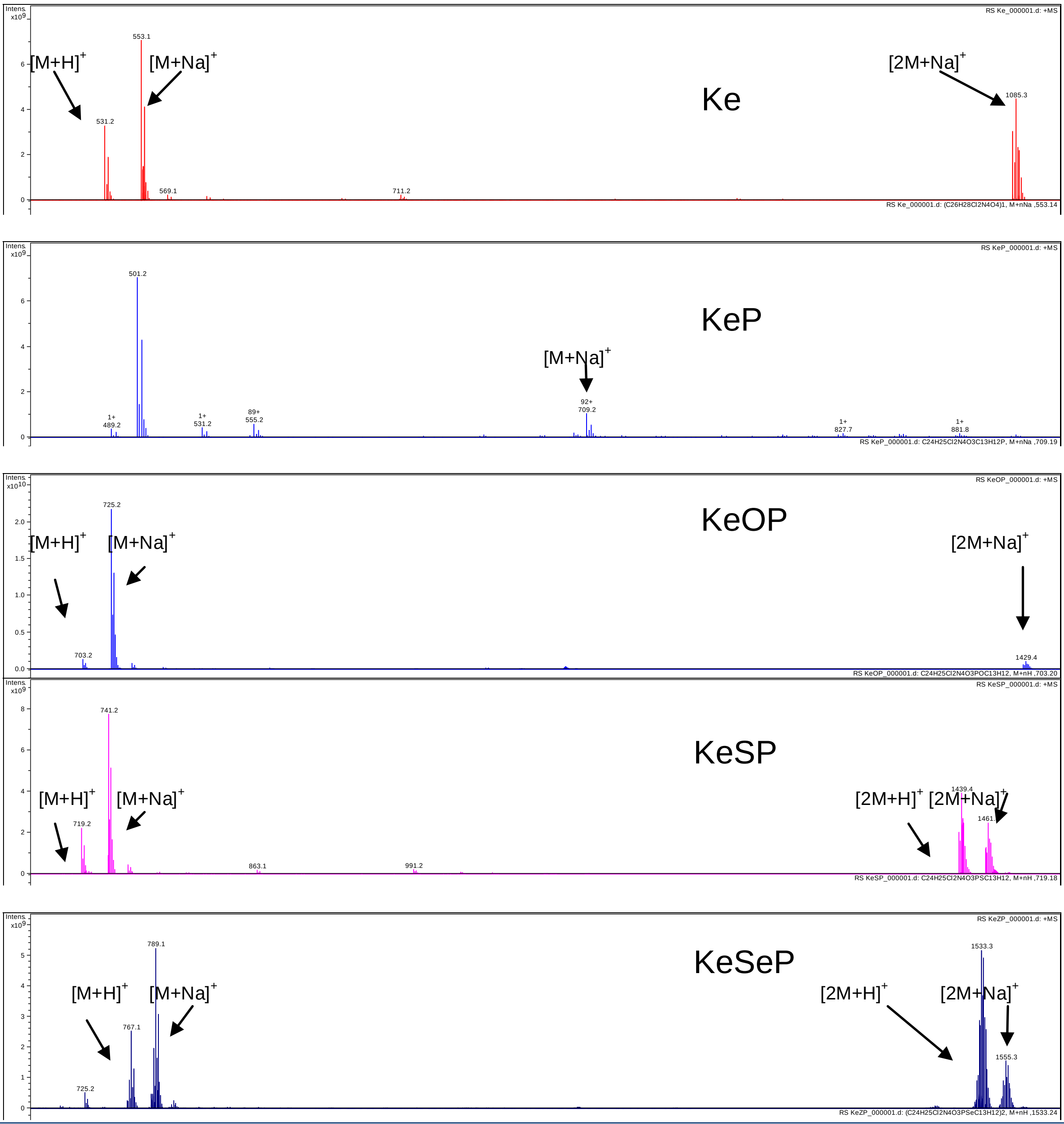

Fig.S2 ESI MS(+) spectra of **Ke**, **KeP**, **KeOP**, **KeSP** and **KeSeP** (from $CHCl_3$ solution).

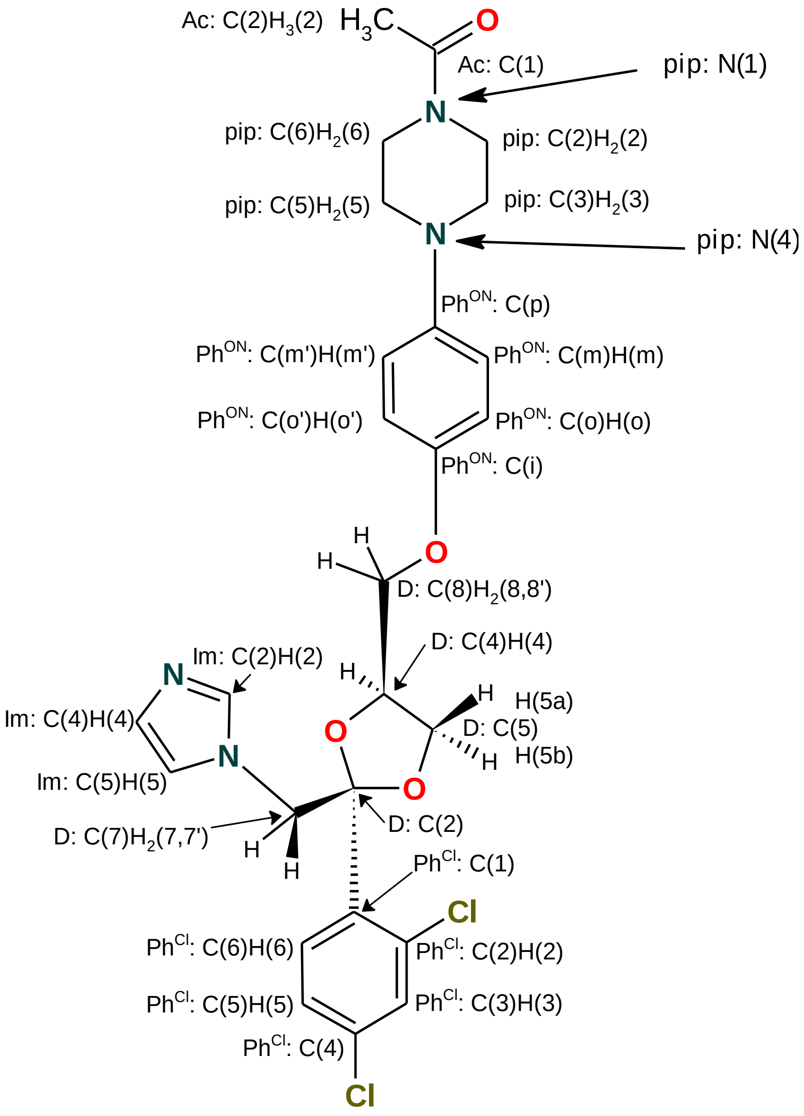

Fig.S3 Molecular scheme of (2S,4R)-(-)-ketoconazole (**Ke**) with atoms numeration.

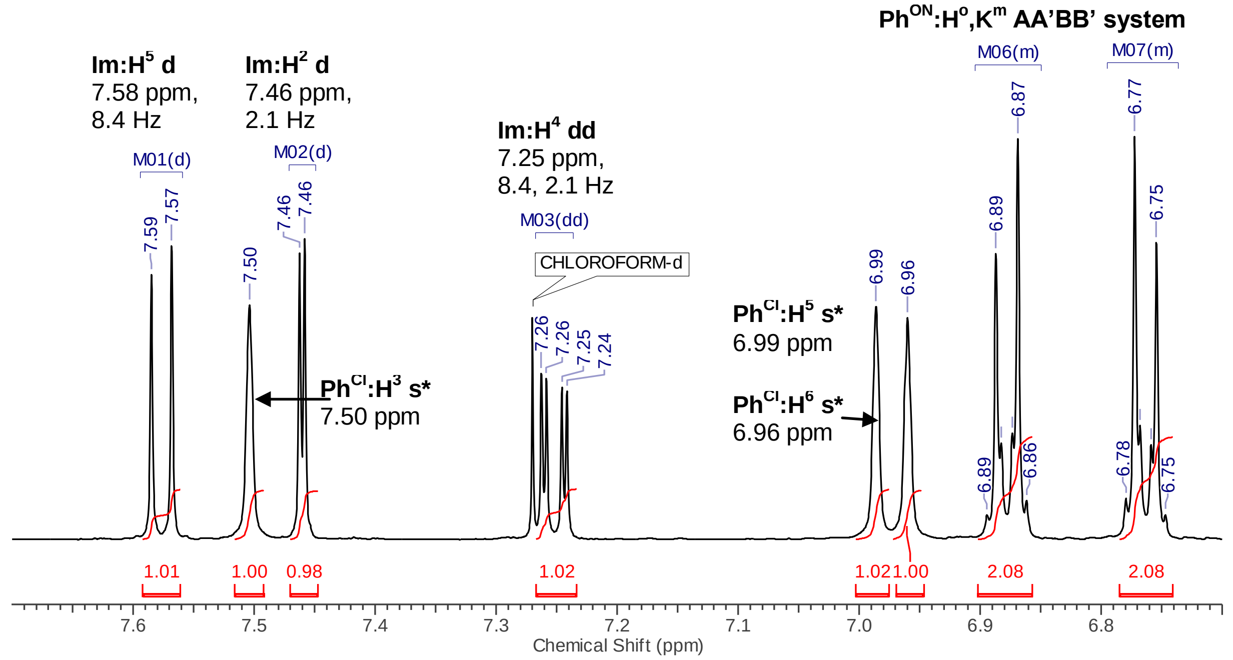

Fig.S4 The 6.7-7.7 ppm fragment of the ^{1}H NMR spectrum of **Ke** in $CDCl_3$ (RS015) and MeOH-d_4 (RS018) with the peaks assignment (atom numeration from Fig.S3).

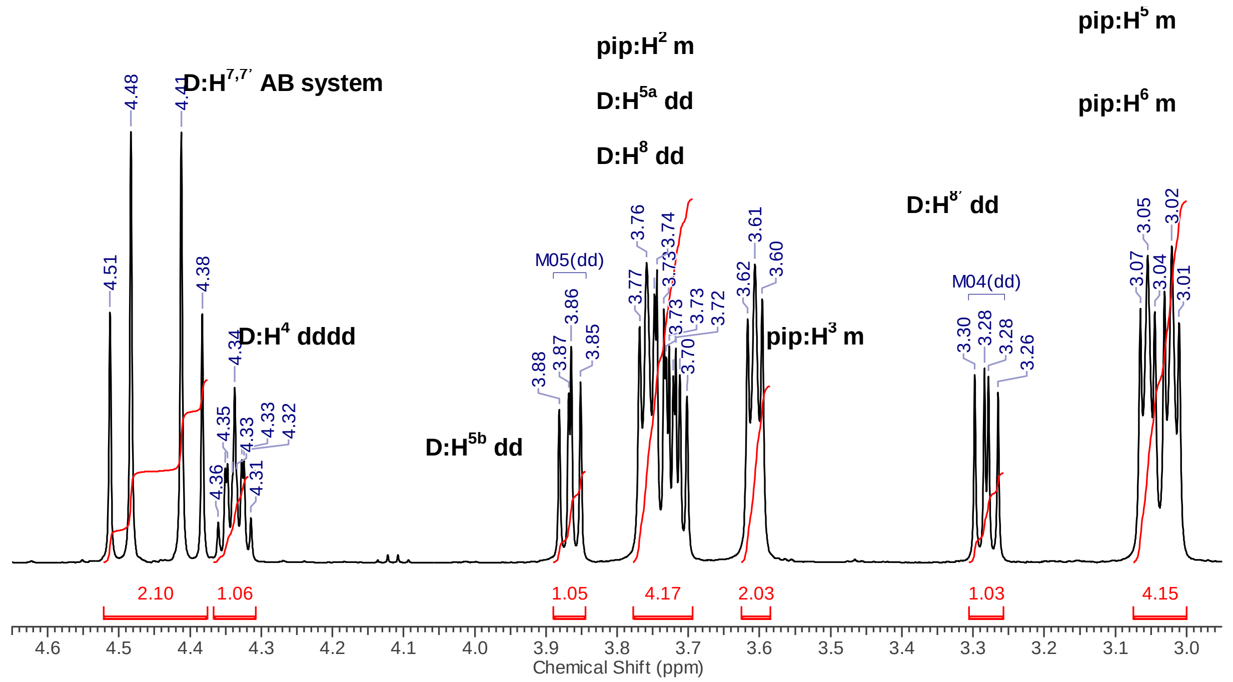

Fig.S5 The 2.95-4.65 ppm fragment of the ^{1}H NMR spectrum of **Ke** in $CDCl_3$ with the peaks assignment (atom numeration from Fig.S3).

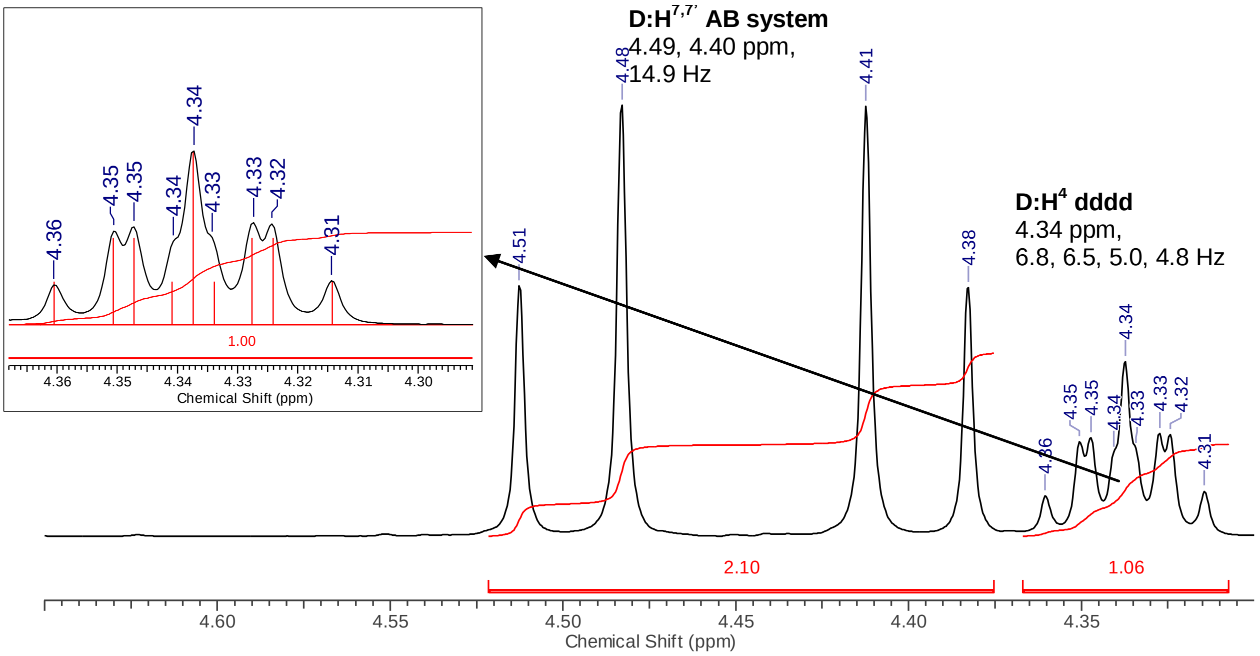

Fig.S6 The 4.30-4.65 ppm fragment of the ^{1}H NMR spectrum of **Ke** in $CDCl_3$ with the peaks assignment (atom numeration from Fig.S3).

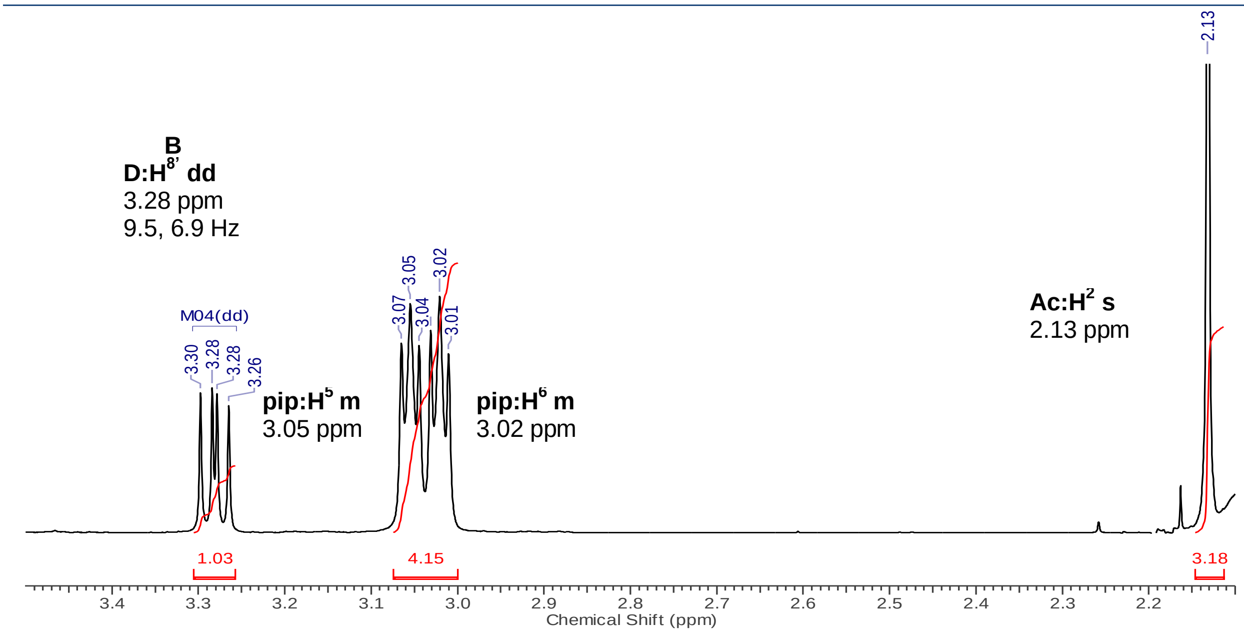

Fig.S7 The 2.10-3.50 ppm fragment of the ^{1}H NMR spectrum of **Ke** in $CDCl_3$ with the peaks assignment (atom numeration from Fig.S3).

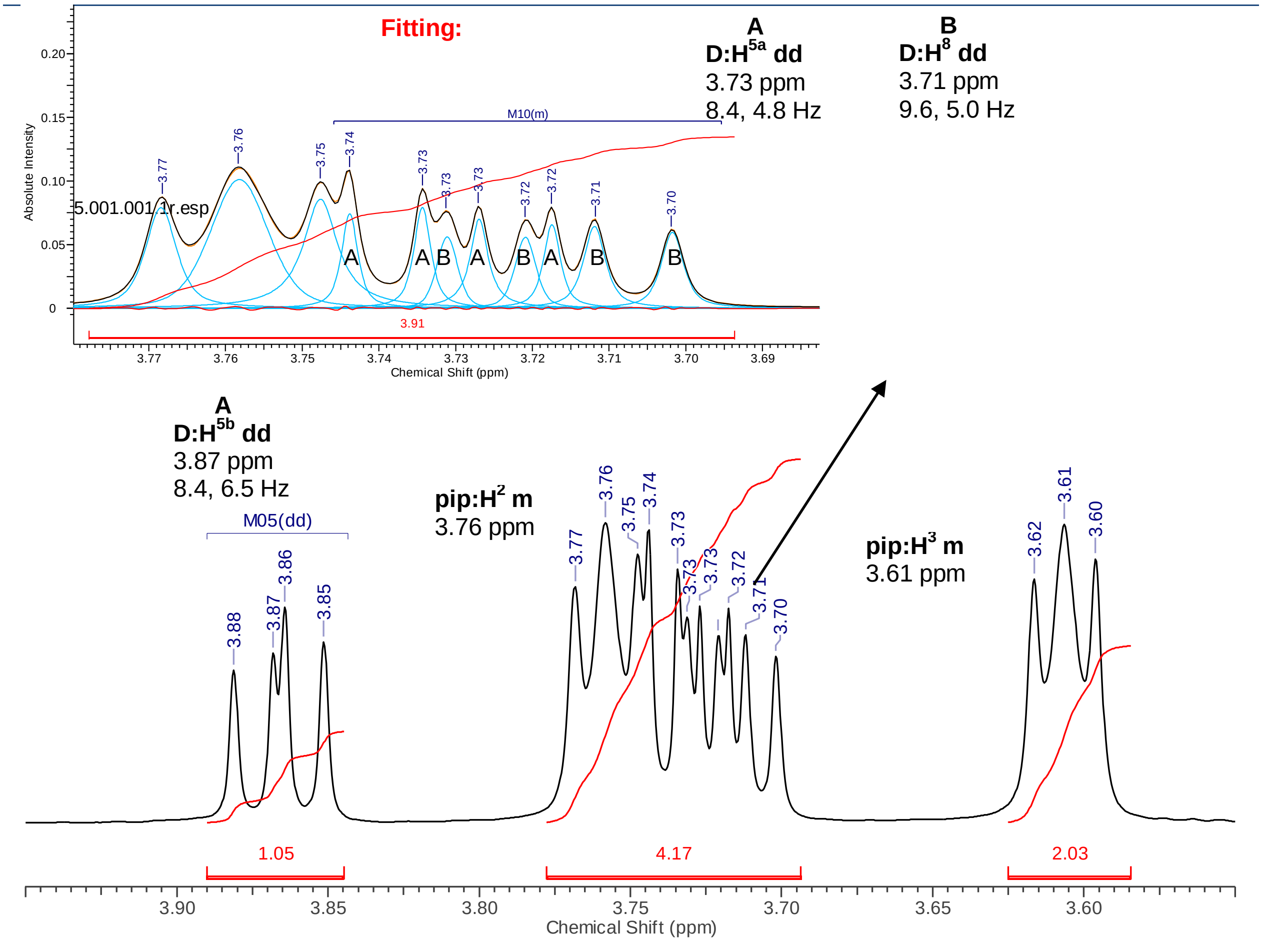

Fig.S8 The 3.55-3.95 ppm fragment of the ^{1}H NMR spectrum of **Ke** in $CDCl_3$ (atom numeration from Fig.S3).

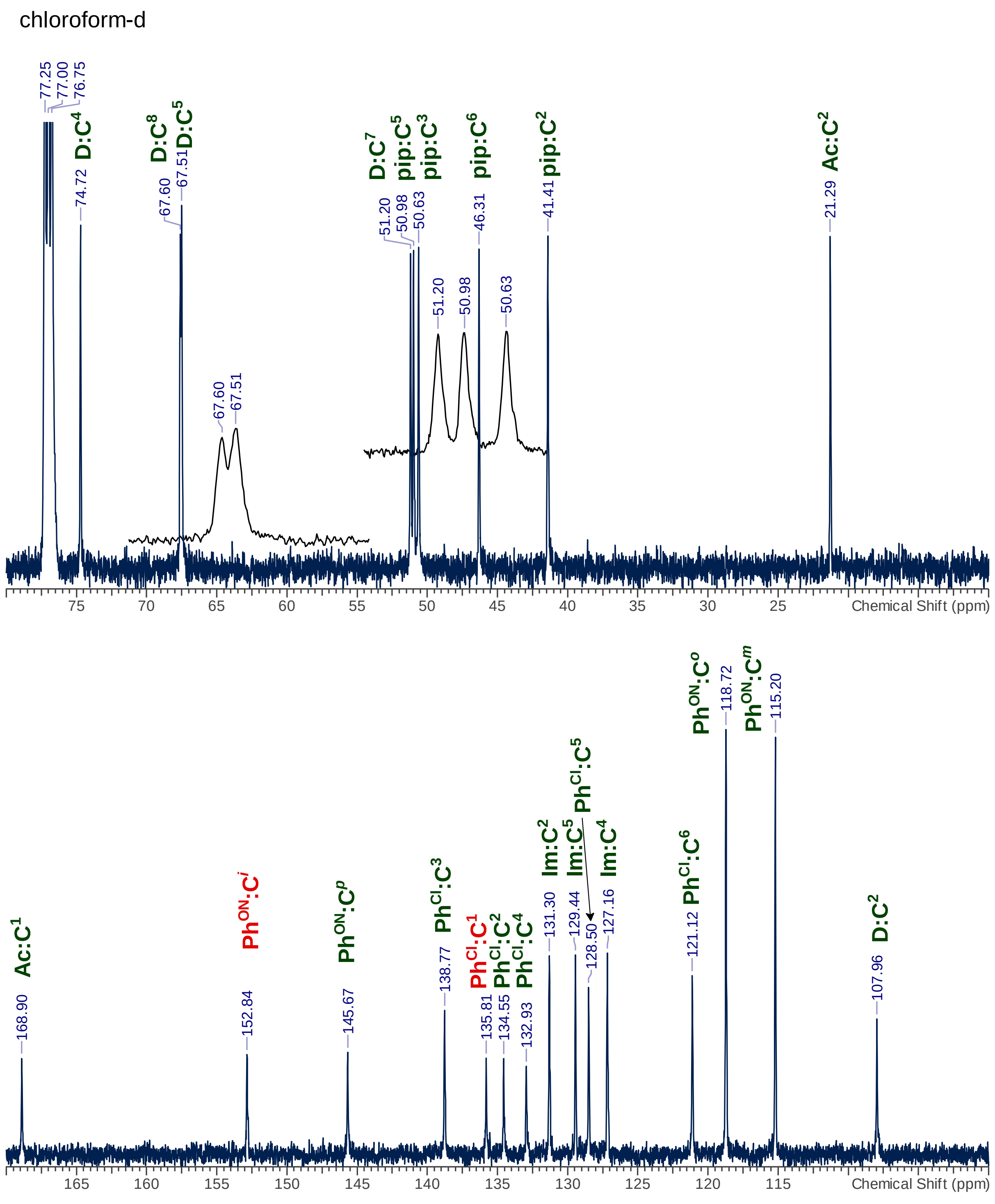

Fig.S9 $^{13}C\{^{1}H\}$ NMR spectrum of **Ke** in $CDCl_3$ with the peaks assignment (atom numeration from Fig.S3).

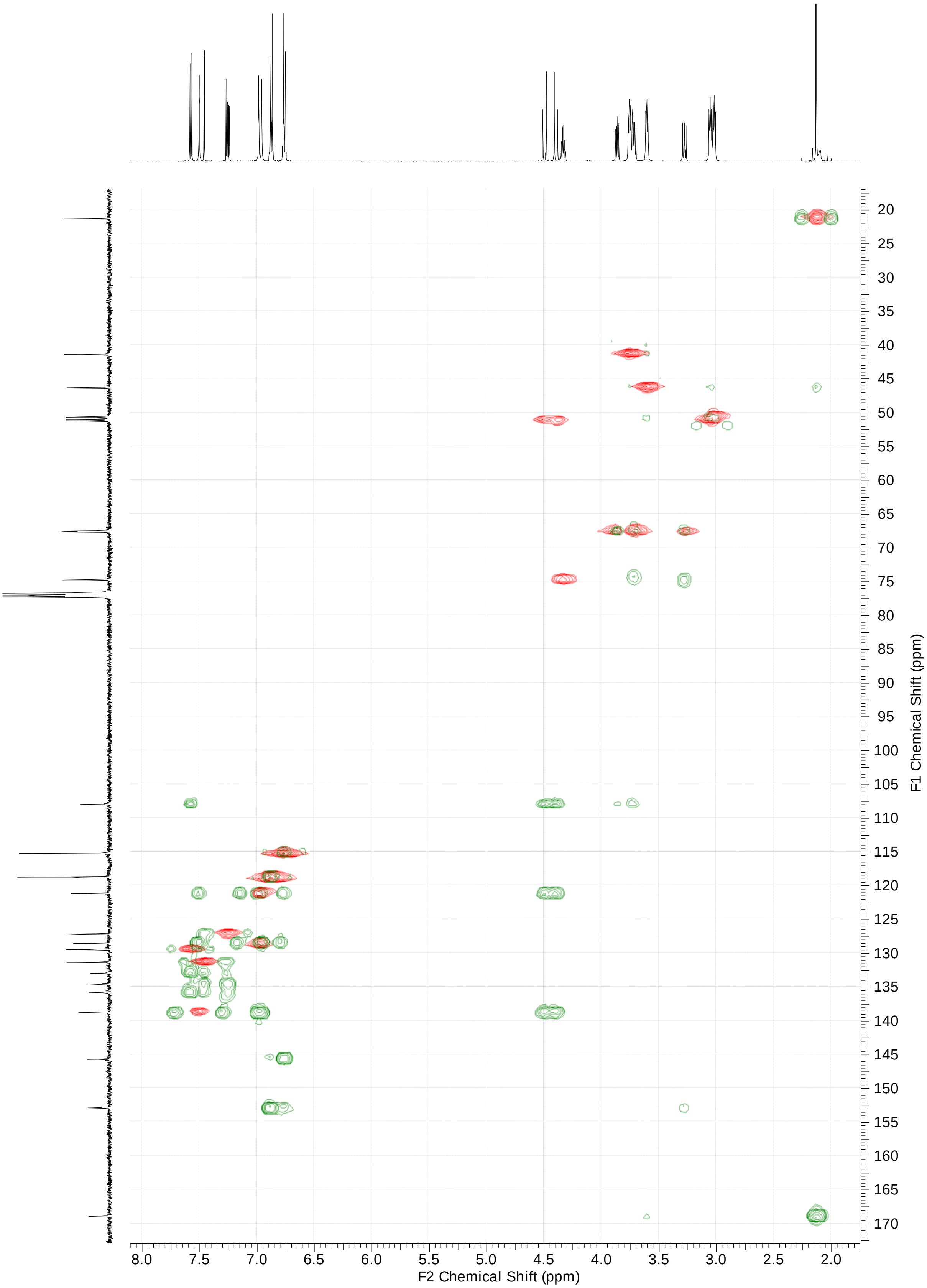

Fig.S10 HMQC (red) and HMBC (green) NMR spectra of **Ke** in $CDCl_3$.

Tab.S1 Chemical shifts [ppm] and coupling constants {Hz] from the NMR spectra of **Ke** in $CDCl_3$.
(atom numeration from Fig.S3)

	$^{13}C\{^{1}H\}$ NMR:		
Ac	**C(1)**	**168.9**	
	**C(2)**	**21.29**	
pip	C(2;_)	41.41	
	C(_;6)	46.31	
	C(3,_)	50.63	
	C(_,5)	50.89	
PhON	C(i)	152.84	
	C(o)	118.72	
	C(m)	115.20	
	C(p)	145.67	
D	C(2)	107.96	
	C(4)	74.72	
	C(5)	67.51	
	C(7)	51.20	
	C(8)	67.70	
Im	**C(2)**	**131.30**	
	**C(4)**	**127.16**	
	**C(5)**	**129.40**	
PhCl	C(1)	135.81	
	C(2)	134.55	
	C(3)	138.77	
	C(4)	132.93	
	C(5)	128.50	
	C(6)	121.12	
	^{1}H NMR:		
Ac	**H(2)**	**2.13**	
pip	H(2;_) m	3.76	
	H(_;6) m	3.61	
	H(3,_) m	3.02	
	H(_,5) m	3.05	
PhON	H(o) AA'	6.88	
	H(m) BB'	6.76	
D	H(4) dddd	4.34	(6.8; 6.5; 5.0; 4.8)
	H(5a) dd	3.73	(8.4; 4.8)
	H(5b) dd	3.87	(8.4; 6.5)
	H(7) A	4.50	(14.88)
	H(7') B	4.40	(14.88)
	H(8) dd	3.71	(9.6; 5.0)
	H(8') dd	3.28	(9.5; 6.9)
Im	**H(2) d**	**7.46**	**(2.1)**
	**H(4) dd**	**7.25**	**(8.6; 2.1)**
	**H(5) d**	**7.58**	**(8.4)**
PhCl	H(3) s*	6.99	
	H(5) s*	6.96	
	H(6) s*	7.50	

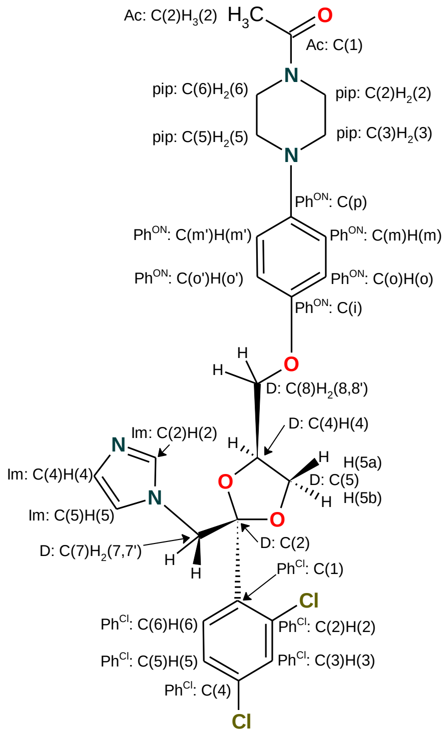

Tab.S2 Chemical shifts and coupling constants (full data) from the carbon, phosphorus and proton NMR spectra of **Ke** and its derivatives in $CDCl_3$. (atom numeration from Fig.S3)

	$^{13}C\{^1H\}$ NMR	**Ke**	**KeP**		**KeOP**		**KeSP**		**KeSeP**	
Ac	**C(1)**	**168.9**								
	**C(2)**	**21.29**								
$-CH_2PPh_2$	**C(1)**		61.40	3.63	58.43	88.10	63.04	73.57	63.54	66.30
	C(i)		138.38	12.71	132.42	97.18	131.94	77.2	130.56	69.63
	C(o)		132.83	18.16	131.28	9.08	131.91	9.99	132.54	9.99
	C(m)		128.36	6.36	128.50	11.81	128.42	11.81	128.44	11.81
	C(p)		128.52	0	131.86	2.7	131.64	2.7	131.74	2.72
pip	C(2;_)	41.41	54.55	9.08	55.54	8.17	55.24	7.27	55.15	6.36
	C(_;6)	46.31								
	C(3,_)	50.63	50.46		50.43		50.40		50.39	
	C(_,5)	50.89								
PhON	C(i)	152.84	152.19		152.28		152.34		152.36	
	C(o)	118.72	117.99		117.98		117.99		118.02	
	C(m)	115.20	115.15		115.18		115.17		115.18	
	C(p)	145.67	146.18		146.05		145.94		145.91	
D	C(2)	107.96	107.97		108.00		107.99		107.99	
	C(4)	74.72	74.79		74.81		74.80		74.80	
	C(5)	67.51	67.71		67.75		67.73		67.73	
	C(7)	51.20	51.27		51.31		51.29		51.30	
	C(8)	67.70	67.57		67.60		67.57		67.57	
Im	**C(2)**	**131.30**	131.30		131.35		131.36		131.37	
	**C(4)**	**127.16**	127.17		127.21		127.21		127.22	
	**C(5)**	**129.40**	129.48		129.51		129.50		129.50	
PhCl	C(1)	135.81	135.80		135.85		135.85		135.88	
	C(2)	134.55	134.60		134.61		134.59		134.59	
	C(3)	138.77	138.74		138.78		138.77		138.77	
	C(4)	132.93	132.94		132.98		132.98		132.99	
	C(5)	128.50	128.55		128.56*		128.50		128.50*	
	C(6)	121.12	121.07		121.12		121.14		121.16	

	$^{31}P\{^{1}H\}$ NMR:	**Ke**		**KeP**		**KeOP**		**KeSP**		**KeSeP**	
				-27.39		27.18		35.15		25.93	721.5 ($^{1}J_{SeP}$)
	^{1}H NMR:	**Ke**		**KeP**		**KeOP**		**KeSP**		**KeSeP**	
Ac	**H(2)**	**2.13**									
-CH_2PPh_2	**H(1)**			3.28	2.86	3.31	6.94	3.53	5.34	3.66	4.58
	Ph			7.32-7.38<br>7.47-7.50	6H<br>4H	7.46-7.55<br>7.83-7.87		7.46-7.55<br>7.97-8.03		7.46-7.55<br>8.00-8.04	
pip	H(2;_) m	3.76		2.83		2.81		2.69		2.67	
	H(_;6) m	3.61									
	H(3,_) m	3.02		3.12		3.03		2.97		2.96	
	H(_,5) m	3.05									
PhON	H(o) AA'	6.89		6.89		6.84		6.82		6.82	
	H(m) BB'	6.77		6.75		6.73		6.73		6.72	
D	H(4) dddd	4.34	(6.8; 6.5; 5.0; 4.8)	4.34	(6.6; 6.5; 5.1; 5.0)	4.34	(6.6; 6.5; 5.1; 5.0)	4.34	(6.6; 6.5; 5.1; 5.0)	4.34	(6.6; 6.5; 5.1; 5.0)
	H(5a) dd	3.73	(8.4; 4.8)	3.74	(8.4; 4.9)	7.73	(8.4, 4.9)	7.73	(8.5, 4.8)	7.73	(8.5, 4.8)
	H(5b) dd	3.87	(8.4; 6.5)	3.88	(8.3; 6.6)	3.87	(8.4; 6.5)	3.87	(8.3; 6.6)	3.87	(8.4; 6.5)
	H(7) A	4.50	(14.88)	4.51	(14.68)	4.51	(14.88)	4.51	(14.88)	4.51	(14.88)
	H(7') B	4.40	(14.88)	4.41	(14.68)	4.42	(14.88)	4.42	(14.88)	4.42	(14.88)
	H(8) dd	3.71	(9.6; 5.0)	3.74	(9.7; 5.1)	3.74	(9.6, 5.1)	3.73	(9.6, 5.1)	3.71	(9.6, 5.1)
	H(8') dd	3.28	(9.5; 6.9)	3.33	(9.6; 6.7)	3.32	(9.6, 6.8)	3.31	(9.6; 6.8)	3.30	(9.7; 6.7)
Im	**H(2) d**	**7.46**	**(2.1)**	**under Ph**		**under Ph**		**under Ph**		**under Ph**	
	**H(4) dd**	**7.25**	**(8.6; 2.1)**	**7.25**	**(8.4; 2.1)**	**7.25**	**(8.4; 2.2)**	**7.26**	**(8.4; 2.1)**	**7.26**	**(8.4; 2.1)**
	**H(5) d**	**7.58**	**(8.4)**	**7.58**	**(8.4)**	**7.58**	**(8.4)**	**7.58**	**(8.4)**	**7.58**	**(8.4)**
PhCl	H(3) s*	6.99		7.00		6.99		6.99		6.99	
	H(5) s*	6.96		6.97		6.96		6.96		6.96	
	H(6) s*	7.50		7.51		**under Ph**		**under Ph**		**under Ph**	

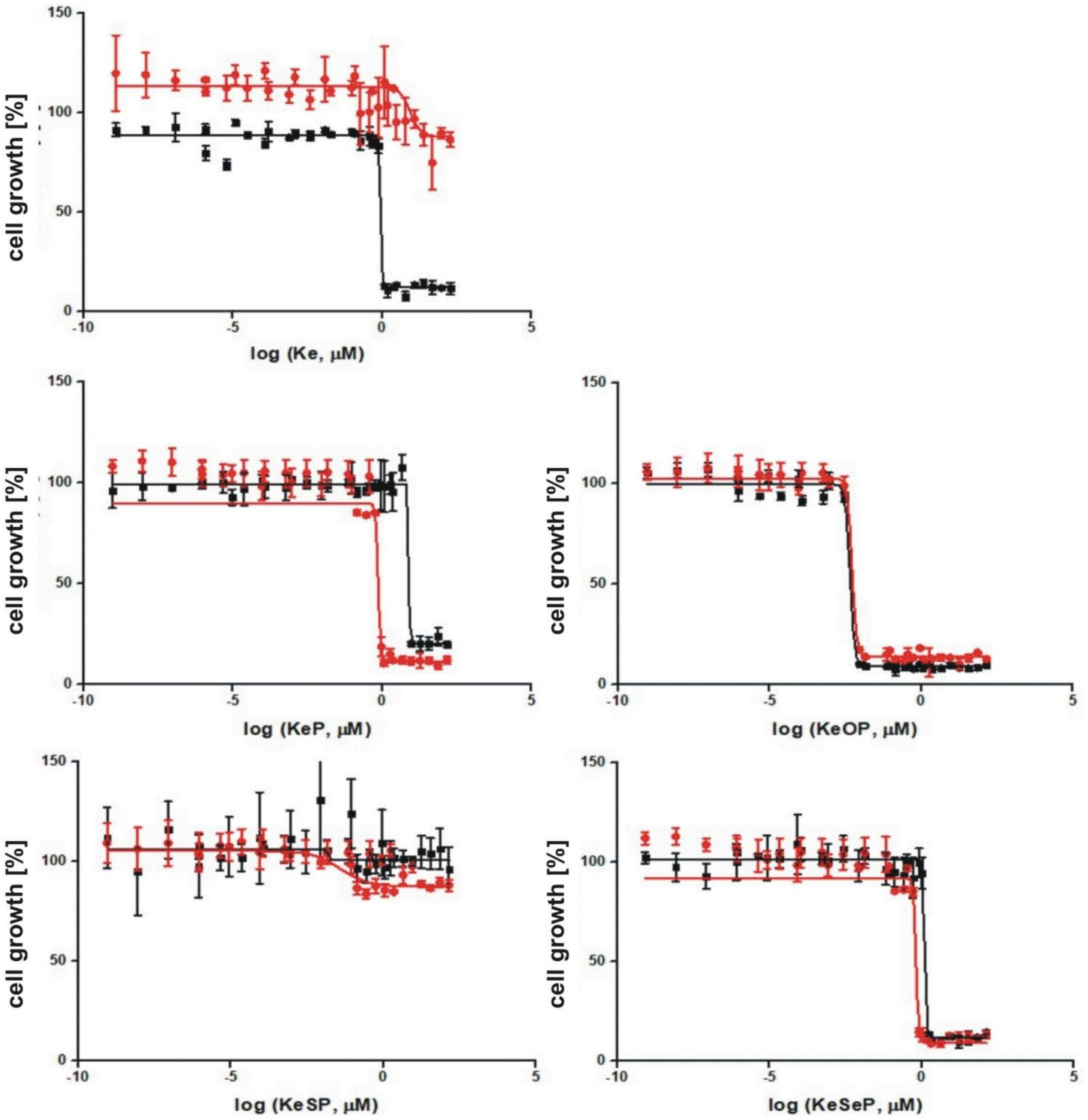

Fig.S11 *S. cerevisiae* wt (black) and *erg6*Δ (red) growth inhibition in the presence of the studied compounds. Percentage cell growth is plotted against the logarithm of treatment concentrations in µM. It was not possible to determine the MIC_{50} value for **KeSP**, and in the case of *erg6*Δ it was not possible to determine the MIC_{50} value for **Ke**.

Tab.S3 KeP/KeOP synergism with **Flc**. Viability [%] of *C. albicans* CAF2-1. Statistical analysis was performed comparing viability at corresponding **Flc** concentrations with/without **KeOP/KeP** (* 0.01 < P < 0.05; ** 0.001 < P < 0.01; *** P < 0.0001). ±SD, n=3

**Flc [µM]**		**0**	**0.78**	**1.56**	**3.13**	**6.25**
	0	100 ± 4.48	86.94 ± 7.84	71.35 ± 13.94	73.85 ± 4.35	37.99 ± 8.94
**KeOP [µM]**	0.1	102.18 ± 2.22	84.62 ± 7.26	86.97 ± 12.74	61.89 ± 8.16	16.38 ± 3.65
	0.2	93.93 ± 7.22	62.2 ± 18.77	56.29 ± 13.73	55.87 ± 18.43	13.09 ± 6.22*
	0.39	91.2 ± 10.66	70.97 ± 12.08	17.89 ± 13.6*	0 ± 12.12**	0 ± 7.32*
**KeP [µM]**	0.39	114.94 ± 9.06	82.35 ± 4.89	70.35 ± 3.98	55.69 ± 4.27*	33.18 ± 8.69
	0.78	101.89± 1.98	71.98 ± 5.97	55.32 ± 10.76*	59.7 ± 3.19*	38.36 ± 10.77
	1.56	96.76 ± 3.21	49.28 ± 14.1*	21.39 ± 13.95*	0 ± 8.36***	0 ± 14.17**

Tab.S4 Sequence of primers used in qRT-PCR

Gene	Sequence 5'→3'	Amplicon length (bp)	Accession number
p53	F: AGATAGCGATGGTCTGGC<br>R: TTGGGCAGTGCTCGCTTAGT	381	NM_001126118.1
p21	F: AGAAGAGGCTGGTGGCTATTT<br>R: CCCGCCATTAGCGCATCAC	169	NM_001220777.1
BAX	F: ACCAAGAAGCTGAGCGAGTGTC<br>R: ACAAAGATGGTCACGGTCTGCC	365	XM_011527191.1
BCl-2	F: ATCGCCCTGTGGATGACTGAG<br>R: CAGCCAGGAGAAATCAAACAGAGG	129	XM_011527191.1
GAPDH	F: GTCAGTGGTGGACCTGACCT<br>R: CACCACCCTGTTGCTGTAGC	256	NM_001289746.1

F: sense primer; R: antisense primer; bp: base pair. p53: tumor suppressor p53; p21: cyclin dependent kinase inhibitor 1A; BAX: Bcl-2 associated X protein; GAPDH: glyceraldehyde 3-phosphate dehydrogenase
